# Integrated Metabolome and Transcriptome Analysis of Fruit Flavor and Carotenoids Biosynthesis Differences Between Mature-Green and Tree-Ripe of cv. “Golden Phoenix” Mangoes (*Mangifera indica* L.)

**DOI:** 10.3389/fpls.2022.816492

**Published:** 2022-02-24

**Authors:** Lei Peng, Wenke Gao, Miaoyu Song, Minghai Li, Dinan He, Ziran Wang

**Affiliations:** ^1^College of Horticulture and Landscape, Yunnan Agricultural University, Kunming, China; ^2^College of Horticulture, China Agricultural University, Beijing, China

**Keywords:** mango (*Mangifera indica* L.), carotenoid biosynthesis, metabolome, transcriptome, flavor, mature-green, tree-ripe

## Abstract

The commodity value of fruits is directly affected by fruit flavor and color. Secondary metabolites, such as amino acids, organic acids, esters, and β-carotene, are important synthetic products, which are of great significance in the flavor formation of mango fruits. In this study, a total of 309 different metabolites, consisting of organic acids, amino acids, phenolic acids, and saccharides, and a further 84 types of volatile organic compounds (VOCs) were identified in differential levels in TR vs. MG mango fruit stages. The major volatile compounds found were ester [2(3H)-furanone, 5-ethyldihydro; N-(2,5-ditrifluoromethylbenzoyl)-D-alanine, pentyl ester; and Octanoic acid, ethyl ester], aldehyde (benzaldehyde, 3-ethyl, and nonanal), and phenol [2-(1,1-dimethylethyl)-6-(1-methylethyl) phenol]. The analysis of carotenoid contents identified 68 carotenoids and we report for the first-time significant contents of zeaxanthin palmitate and (E/Z)-phytoene in mango fruits. α-carotene was a further major contributor to carotene contents with lesser contributions from 5,6epoxy-lutein-caprate-palmitate, β-carotene, lutein oleate, and β-cryptoxanthin. What is more, lutein content was significantly decreased in TR vs. MG fruit. RT-qPCR analysis revealed that relative to the MG stage, the expression of carotenogenic genes GGPS, PSY, LCYB, and ZEP was downregulated in TR mango fruit, whereas the transcript levels of PSD, CHYB, and NCED were downregulated. Additionally, the transcription level of some transcription factors (MYB, bHLH, and NAC) was highly correlated with pigment content in the pulp and may be responsible for carotenoid accumulation. The results describe major differences in metabolic pathways during the transition from MG to the TR stage of fruit ripening that are likely to contribute alterations in fruit flavor and provide several associated genes to be further studied in mango fruit.

## Introduction

Mango (*Mangifera indica* L.), known as the “king of tropical fruits,” is a world-renowned tropical fruit originating from India. Different types of mango fruits can vary largely in peel and pulp colors, shape, and aroma. In recent years, the land area used for mango cultivation has increased and currently, mango ranks third among tropical fruits and has become one of the top five fruits in the world (Galán Saúco, 2017). Mangoes contain many healthy and beneficial bioactive components including rich dietary fibers, minerals, vitamin C, carotenoids, and polyphenols in addition to their provision of sugar and energy (Ma et al., 2017). Mangoes can be generally divided into those with red skin and yellow pulp, green skin and yellow pulp or yellow skin, and yellow pulp. Fruit color is a vital index for the evaluation of the quality and commodity value of fresh fruits, and bright and attractive fruit colors are important factors affecting the choice of both growers and consumers (Zhou et al., 2015; Wang et al., 2017b).

The flavor of fruits is also a vital factor affecting consumer choice. Both fruit aroma and taste contribute to the formation of fruit flavor where aroma arises from volatile compounds, such as phenols and alcohols, whereas taste is determined by the ratio and concentration of saccharides, organic acids, and amino acids (Zou et al., 2020). In mangoes, the aroma is a crucial sign of mango fruit ripening and is unique to each variety. Currently, over 270 free aromatic volatiles have been identified in mango varieties (Herianus et al., 2003; Lalel et al., 2003), some of which can be released after hydrolysis of their glycosides during fruit storage (Sakho et al., 1997; Ubeda et al., 2012). In recent years, the recognition of the importance of volatile aromatic compounds and their contribution to fruit aroma has motivated an increasing number of studies (Munafo et al., 2014). Secondary metabolites, such as volatile metabolites, are also present in different fruits, including passion fruit (Xia et al., 2021), loquat (Zou et al., 2020), papaya (Pino et al., 2003), apricot (Jiang et al., 2019), and yellow peach (Raffo et al., 2008). Previous studies in mango fruit have mainly focused on color, soluble solids, total sugar, and total acid composition and little research has been undertaken into the changes in the transcription and metabolic networks between mature-green and tree-ripe stages of mango fruits. Moreover, the changes in flavor substances and carotenoid contents in the transition from mature-green to tree-ripe mango fruits have not been systematically and comprehensively studied.

As a class of yellow, orange-red, or red polyenes, carotenoids are generally composed of eight isoprenoid units, which can resist oxidation and prevent cancer and night blindness (Krinsky, 2006). In plants, carotenoids act as auxiliary pigments for photosynthesis in chloroplasts, protect chlorophyll against damage from strong light, and also serve as precursors for abscisic acid (ABA) synthesis (Moreno et al., 2020). The biosynthesis pathway of carotenoids has been studied intensely. However, isopentenyl/dimethylallyl diphosphate isomerase (IPI) and geranylgeranyl diphosphate synthase (GGPS) lead to the conversion of isopentyl diphosphate dimethylallyl diphosphate and the production of geranylgeranyl diphosphate (GGPP), respectively. The GGPP carotenoid precursor is used in other metabolic pathways, including chlorophyll and tocopherol synthesis. The production of phytoene from geranylgeranyl diphosphate (GGPP) by phytoene synthase (PSY) is the first committed step in carotenoid biosynthesis. As a crucial synthetic product in the carotenoid-biosynthesis pathway, β-carotene accounts for 60–70% of the total carotene content (Jiang et al., 2019) and provides ripe mango fruits with a distinctive orange color (Ellong et al., 2015). As the main precursor of vitamin A, β-carotene is also one of the most important functional components in fruit development (Vásquez-Caicedo et al., 2005). Vitamin A, an essential nutrient for humans, cannot be synthesized in the human body. Mangoes are nutritional and have certain pharmacological significance due to their high antioxidant content (Núñez-Sellés et al., 2007). The high content of β-carotene in mangoes is considered a dietary supplement and also a potential source of functional ingredients in processed foods and is used for the control, management, and treatment of oxidative stress-induced health problems (Onuh et al., 2017).

Studies have demonstrated that the expression of genes involved in β-carotene biosynthesis is regulated by various transcription factors (TFs). TFs are roughly divided into families, such as MYB, bHLH, MADS-box, and basic leucine zipper (bZIP) based on their different conserved domains (Lu et al., 2021). The TF CrMYB68 can lead to the accumulation of tangerine carotenoids by directly and negatively regulating CrBCH2 and CrNCED5 (Zhu et al., 2017). SlMYB72 is able to modulate the biosynthesis of tomato carotenoids by directly binding to PSY, ZDS, and LCYB enzyme genes (Wu et al., 2020). CpbHLH1 and CpbHLH2 individually modulate the transcription of LCYB genes (CpCYC-B and CpLCY-B) during papaya fruit ripening (Zhou et al., 2019). However, the regulatory mechanisms underlying β-carotenoid biosynthesis and metabolism in mangoes have been scarcely studied. A greater understanding of these regulatory mechanisms in mangoes would lay the foundation for cultivating mango with beneficially higher β-carotene content.

In this study, transcriptomics and metabolomics of volatiles and carotenoids were used to examine mango fruits at the mature-green (MG) and tree-ripe (TR) stages of fruit development for their differences in fruit flavor and carotenoid contents. In addition, the differences in related primary metabolites including carbohydrates, organic acids, amino acids, esters, alcohols, terpenoids, and heterocyclic compounds were characterized. Furthermore, the differential changes in related TFs (MYB, bHLH, and NAC) and principal genes (GGPS, PSY, and LCYB) of the carotenoid-biosynthesis pathways were analyzed. The aim of this study was to investigate the effects of mature-green and tree-ripe mango fruit flavor and to provide new insights into the changes in metabolic and transcriptional regulatory networks underlying the development of appearance, taste, and aroma qualities in mango fruit. Our study lays the groundwork for further elucidation of crucial players in mango pigmentation and flavor and to offer a theoretical basis for providing a reference for the production of high-quality mangoes.

## Materials and Methods

### Plant Materials and Treatment

The Early-Mid Ripe mango cultivar “Golden Phoenix” was planted in Yuanjiang Hani, Yi, and Dai Autonomous County (101°39′-102°22′N, 23°18′-23°55′W) of Yuxi City, Yunnan Province, China. Mango materials were provided by Yuanjiang County Agricultural Technology Extension Service Center in Yunnan province. Experimental research on mangoes was conducted in compliance with China laws and regulations and local legislations in Yunnan province. The mango trees were 8 years old with a row spacing and plant spacing of 3 × 5 m, respectively. MG fruits were harvested 120 days after flowering (reaching physiological maturity), and TR fruit was harvested about 150 days after flowering in the summer of 2021. According to the project listed by Supplementary Table S1, their fruit quality was measured as follows: transverse and longitudinal diameters of the fruit were measured with a Vernier caliper, and the fruit shape index was the ratio of the longitudinal to transverse diameters. Fruit texture was measured using a durometer (Fujiwara FHM-1, Japan). Fruit total soluble solids content was measured with a hand-held refractometer (ATAGO PAL-1, Japan). Titratable acid content was determined by NaOH titration. Excel 2016 was used for data sorting and Origin 8.5 for chart drawing. Correlation analysis was performed using SPSS 19.0 (SPSS Inc., Chicago, IL, United States). Data from all analyses were expressed as average and standard error. The threshold of significance was set at *p* < 0.05. Each group consisted of 20 fruits, in triplicate, and samples were designated MG 1, 2, 3 and TR 1, 2, 3, respectively. Three biological replicates were set for each sample, and 20 fruits were collected randomly from five trees of the same growth in each replicate. After taking mangos back to the laboratory, the middle part of pulps (about 15 g in weight) was carefully excised with a razor blade, immediately frozen in liquid nitrogen, and stored at-80°C for further analysis.

### Sample Preparation Has Been Metabolite Extraction, Identification, and Quantification

Mango fruit samples were well ground in liquid nitrogen and crushed at 30 Hz for 1.5 min using a hybrid mill with zirconia beads (MM400, Retsch). Powdered samples (100 mg) were weighed and extracted overnight at 4°C with 1.0 ml of 70% aqueous methanol. Following centrifugation at 10,000 rpm for 10 min, the extracts were filtrated (SCAA-104, 0.22 μm pore size; ANPEL, Shanghai, China)^1^ for UPLC–MS/MS analysis. The metabolite extraction, identification, and quantification were performed previously (Wang et al., 2017a).

The sample extracts were analyzed using an UPLC-ESI-MS/MS system (UPLC, SHIMADZU Nexera X2,^2^ MS, Applied Biosystems 4,500 Q TRAP).^3^ The analytical conditions were as follows, UPLC: column, Agilent SB-C18 (1.8 μm, 2.1 mm*100 mm); the mobile phase was consisted of solvent A, pure water with 0.1% formic acid, and solvent B, acetonitrile with 0.1% formic acid. Sample measurements were performed with a gradient program that employed the starting conditions of 95% A, 5% B. Within 9 min, a linear gradient to 5% A, 95% B was programmed, and a composition of 5% A, 95% B was kept for 1 min. Subsequently, a composition of 95% A, 5.0% B was adjusted within 1.10 min and kept for 2.9 min. The column oven was set to 40°C; the injection volume was 4 μl. The effluent was alternatively connected to an ESI-triple quadrupole-linear ion trap (Q TRAP)-MS (AB SCIEX, United States). Triple quadrupole-linear ion trap mass spectrometer (QTRAP; API 4500 Q TRAP LC/MS/MS System) was used for Linear Ion Trap (LIT) and triple quadrupole (QQQ) scans. QQQ and LIT modes with 10 and 100 μmol/l polypropylene glycol solutions, respectively. Metabolite data analysis and quantification were performed using Analyst 1.6.1 software (AB SCIEX, Ontario, Canada) and multiple reaction monitoring (MRM), respectively. Finally, the identified metabolites were subjected to partial least squares discriminant analysis (PLS-DA). Significantly regulated metabolites between groups were determined by VIP ≥ 1, *value of p* < 0.05, and absolute Log2FC (fold change) ≥ 1. VIP values were extracted from OPLS-DA result, which also contain score plots and permutation plots, was generated using R package MetaboAnalystR. The data were log transform (log2) and mean centering before OPLS-DA. In order to avoid overfitting, a permutation test (200 permutations) was performed (Zhang et al., 2020).

### Isolation, Concentration, and Identification of Volatiles

Mango samples were ground into a powder in liquid nitrogen. 1 g of the mango powder was weighed and transferred immediately to a 20 ml headspace vial (Agilent, Palo Alto, CA, United States), containing NaCl saturated solution to inhibit any enzyme reaction. The vial was sealed using a crimp-top cap with TFE-silicone headspace septa (Agilent). At the time of solid-phase microextraction (SPME) analysis, the vial was placed in 100°C for 5 min; then, a 120 μm divinylbenzene/carboxen/polydimethylsiloxane fiber (Agilent) was exposed to the headspace of the sample for 15 min at 100°C. After sampling, desorption of the VOCs from the fiber coating was carried out in the injection port of the GC apparatus (Model 8,890; Agilent) at 250°C for 5 min in the splitless mode. The identification and quantification of VOCs were carried out using an Agilent Model 8,890 GC and a 5977B mass spectrometer (Agilent), equipped with a 30 m × 0.25 mm × 0.25 μm DB-5MS (5% phenyl-polymethylsiloxane) capillary column. Helium was used as the carrier gas at a linear velocity of 1.2 ml/min. The injector temperature was kept at 250°C and the detector temperature at 280°C. The oven temperature was programmed from 40°C (3.5 min), increasing at 10°C/min to 100°C, at 7°C/min to 180°C, and at 25°C/min to 280°C, hold for 5 min. Mass spectra were recorded in electron impact (EI) ionization mode at 70 eV. The quadrupole mass detector, ion source, and transfer line temperatures were set, respectively, at 150, 230 and 280°C. Mass spectra were scanned in the range m/z 50–500 amu at 1 s intervals. Identification of volatile compounds was achieved by comparing the mass spectra with the data system library (MWGC or NIST) and linear retention index (Chai et al., 2012; Guo et al., 2016; Xia et al., 2021).

### Detection of Carotenoid Metabolites

The mango pulp was freeze-dried by liquid nitrogen and ground into a powder in a mortar. 50 mg of the dried powder was weighed and put into a mixture of hexane, acetone, and ethanol, followed by addition of the internal standard. The stock solution of the standard was stored at a concentration of 1 mg/ml at-20°C. Next, the extract was vortexed for 20 min at room temperature and centrifuged (10,000 rpm for 10 min, 5424R, Eppendorf, German), and the supernatant was collected, then evaporated to dryness under a stream of nitrogen, and reconstituted in a mixture of methanol and methyl tertiary butyl ether (MTBE). The mixed solution was then filtered through a 0.22 μm filter for further LC–MS analysis. Subsequently, LC- Atmospheric Pressure Chemical Ionization (APCI)-MS/MS (UHPLC, ExionLC™ AD,^4^ MS, Applied Biosystems 6,500 Triple Quadrupole; see footnote 4) was conducted under the following conditions: HPLC column: YMC C30 (3 μm, 100 mm × 2.0 mm i.d), solvent system consisting of methanol, acetonitrile (1: 3, v/v) with 0.01% butylated hydroxytoluene (BHT) and 0.1% formic acid (A), and methyl tert-butyl ether with 0.01% BHT (B), gradient program: 0% B (0–3 min), 70% B (3–5 min), 95% B (5–9 min), and 0% B (11–12 min), flow rate: 0.8 ml/min, temperature: 28°C, and injection volume: 2 μl. A API 6500 + Q TRAP LC/MS/MS system, equipped with an APCI Turbo Ion-Spray interface, was utilized in a positive ion mode and controlled by Analyst 1.6.3 software (AB Sciex). The APCI source operation parameters included ion source: APCI+, source temperature: 350°C, and curtain gas (CUR): 25.0 psi. Following optimization, DP and CE were conducted for individual MRM transition. In the end, a specific set of MRM transitions were monitored during each period according to the carotenoids eluted within this period (Rodrigues et al., 2016; Petry and Mercadante, 2018).

### RNA-Seq and Annotation

A total of six transcriptome sequencing libraries were constructed using two mango pulp samples with three biological replicates each. Total RNA extraction from mango pulps was performed using the TRIzol method (Xia et al., 2021). RNA concentration and purity were determined by NanoDrop 2000 (NanoDrop Technologies, Wilmington, DE, United States) and Agilent Bioanalyzer 2,100 system (Agilent Technologies, Palo Alto, CA, United States), respectively. The integrity of the RNA was determined using 1% agarose gel electrophoresis. Afterward, mRNA was isolated from total RNA using magnetic beads with oligo (dT), and complementary deoxyribonucleic acid (cDNA) was synthesized by ligating sequencing adapters to both ends using a cDNA synthesis kit (TaKaRa, Japan). Next, library preparations were sequenced on the Illumina HiSeq 4,000 platform, and single gene sequences obtained from the Green Plant Transcriptome database were integrated and annotated by RSEM 1.3.1 software (Dewey and Li, 2011).

### Transcriptome Data Analysis

Unigene sequences were compared with KEGG, NR, Swiss-Prot, GO, COG/KOG, TrEMBL databases using BLAST software, and the amino acid sequences of unigenes were predicted and compared with Pfam database using HMMER software to obtain annotation information of unigenes. The unnormalized reads count data of the gene were entered on DESeq2. Next, the Benjamini-Hochberg method was used to correct multiple hypothesis testing probabilities (*value of p*). Differential genes were screened based on |Log_2_Fold Change| ≥ 2 and FDR < 0.05. The fragments per kilobase of exon model per million mapped reads (FPKM) of genes were centralized and normalized, and then, Kmeans clustering analysis was performed. Subsequently, after annotating the genes into the KEGG database, the number of differential genes contained in each KEGG pathway was counted, and the significantly enriched pathway was identified among the differentially expressed genes. Finally, the distribution of differential genes in the Gene Ontology (GO) was investigated using enrichment analysis to elucidate the functional representation of sample differences in genes in the experiment. Plant TF prediction was performed using iTAK 1.7 software,^6^ which integrates two databases, namely, PlnTFDB and PlantTFDB (Zheng et al., 2016).

### RT-qPCR Verification

Total RNA was extracted from mango fruits by the CTAB method (Wang et al., 2019). Based on the transcriptome data of TR vs. MG mango fruits, the expression level of 13 carotenoid-biosynthetic pathway genes and seven TFs (MYB, bHLH, and NAC) was validated. The PCR was performed with an ABI 7500 Fast Real-Time Detection System (Applied Biosystems, Waltham, MA, United States) using the Ultra SYBR Mix Kit (TaKaRa, Dalian, China). In the amplification system (the total volume of 20.0 μl), where there were 10.0 μl of Ultra SYBR Premix System II, 0.5 μl of 10 μM upstream primer, 0.5 μl of 10 μM down-stream primer, 2.0 μl template, and 7.0 μl double-distilled water, the amplification was conducted at 95°C for 8 min, followed by 38 cycles of 95°C for 5 s and 60°C for 30 s. Relative quantitative analysis of data was performed by the 2^−ΔΔCT^ method with *β-*actin as the reference gene. The primers used for RT-qPCR are listed in Supplementary Table S2.

## Results

### Transcriptome Sequencing, Clustering, and Function Enrichment

**Figure 1:**
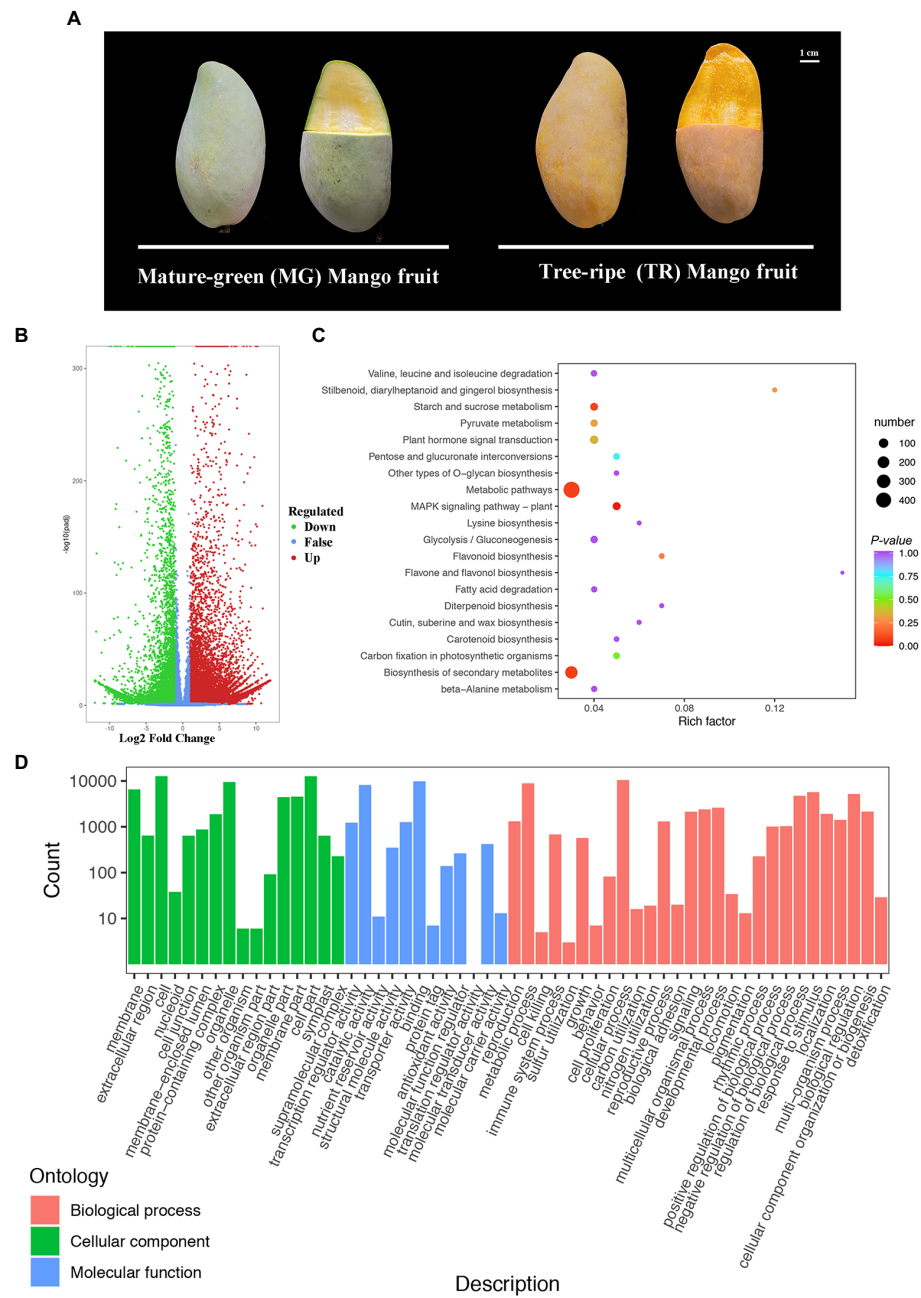
Gene expression patterns and KEGG enrichment analyses during the development of mango fruits (*Mangifera indica* L.). **(A)** Representative images of “Golden Phoenix” mango fruits at different developmental stages for transcriptome. MG: Mature-green mango fruit. TR: Tree-ripe mango fruit. Scale bar = 1 cm. **(B)** Volcano plot of DEGs for TR vs. MG. **(C)** Pathway enrichment analysis of DEGs for TR vs. MG. The color of the point represents *p*, and the size of the point represents the number of enriched DEGs. **(D)** GO classification of unigenes of *Mangifera indica* L. The results are summarized in TR vs. MG.

Mangoes of the cv. “Golden Phoenix” at fruit ripening stages of MG and TR are shown in Figure 1A. MG and TR stages mangoes were sequenced with three biological replicates for each stage. The paired-end reads of cDNA libraries of each were obtained using the Illumina HiSeq 4,000 platform and are shown in Supplementary Table S3. The mapping rates obtained though comparison with the reference database were 84.80 and 89.03% (Supplementary Table S3). Differentially expressed genes (DEGs) identified among the two samples were filtered for an FDR < 0.05 and a Log_2_FC ≥ 2. The data indicated 20,120 DEGs between MG and TR, with the number of upregulated genes slightly larger than that of downregulated genes (12,870 vs. 7,250; Figure 1B). Biological pathways were assigned to the DEGs from the KEGG database after screening for pathways with a *value of p* < 0.05. The results show that the DEGs in “TR vs. MG” were significantly enriched in plant hormone signal transduction, carbon metabolism, pyruvate metabolism, metabolic pathways, and biosynthesis of secondary metabolites (Figure 1C). Substantial and significant enrichment was observed in both metabolic pathways and biosynthesis of secondary metabolites. These metabolic pathways shed light on the metabolic processes occurring during the development of mango fruits. From the GO^7^ annotations of the DEGs, it can be seen that 1,154 unigenes were annotated as “Biological Process,” 674 as “Cellular Component,” and 572 as “Molecular Function” (Figure 1D).

### Metabolomic Analysis of the Differences in Flavor Substances Between MG and TR Mango Fruits and Corresponding Changes in Metabolically Related Gene Transcription

**Figure 2:**
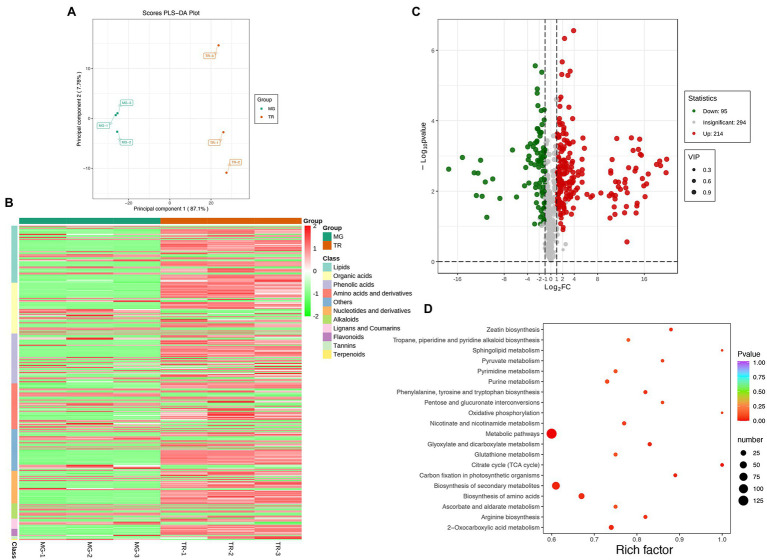
Preliminary analysis of metabolomics data of mango fruits. **(A)** PLS-DA of samples with six biological repetitions at two developmental stages. **(B)** Heat map of different metabolites in MG and TR. **(C)** Volcano plot of differential metabolites for TR vs. MG. Each point in the figure represents a metabolite. Green points represent downregulated metabolites, red points represent upregulated metabolites, and gray points represent metabolites that were detected but not significantly different. **(D)** Pathway enrichment analysis of differential metabolites for TR vs. MG. The color of the point represents *p*, and the size of the point represents the number of differentially enriched metabolites.

To understand the molecular mechanism leading to differences in flavor compounds in the development of “Golden Phoenix” mangoes, a metabolomic comparison of MG and TR stages using high performance liquid chromatography-mass spectrometry (HPLC-MS/MS) was conducted. Partial least squares discriminant analysis (PLS-DA) of the HPLC-MS/MS data showed a clear difference between MG and TR samples along PC1 which accounts for 87.1% of the total variance (Figure 2A). The majority of the residual variance (PC2; 7.76%) appears to be due to differences in biological replicates. Using metabolite concentration data for the cluster analysis of a stratified heat map of the samples, it was observed that all biological replicates were grouped together (top of the figure), which indicates a high reliability of the resulting metabolome data (Figure 2B). The metabolomic analysis revealed that 309 metabolites showed differential levels in MG vs.TR fruits, including organic acids, esters, terpenes, saccharides, and alcohols (Supplementary Table S4). After filtration of these metabolites for those with a Log2FC ≥ 1 or ≤ −1, and a variable importance projection (VIP) ≥ 1, 214 upregulated and 95 downregulated metabolites were identified in TR vs. MG (Figure 2C). The pathways associated with the metabolites thus selected were identified by use of the KEGG database. The results indicated these metabolites were mainly enriched in pathways including metabolic pathways, citrate cycle (TCA cycle), biosynthesis of secondary metabolites, and biosynthesis of amino acids (Figure 2D).

The software package^8^ “Cor” in the language package R was used to calculate the Pearson correlation coefficient (PCC) of DEGs and differential metabolites, with the fold-changes of metabolites with a PCC > 0.8 in each group shown in the nine-quadrant diagram (Supplementary Figure S1). In the third and seventh quadrants, a total of 12,839 DEGs and 71 metabolites were detected in “TR vs. MG.” The consistence in differences in gene expressions and metabolites indicates that in general, the changes in gene expression drive the observed changes in metabolite levels. A two-way orthogonal PLS (O2PLS) model was established for all DEGs and differential metabolites and according to the load chart, variables with high relevance and significant weight in the different datasets were preliminarily evaluated to select important metabolites (Supplementary Figure S2). The nine metabolites most affected by the transcriptome included 2 amino acids and derivatives (Benzyl-(2”-O-xylosyl)glucoside; N-acetyl-L-leucine), 2 nucleotides and derivatives (9-(arabinosyl)hypoxanthine; Guanine), 3 phenolic acids (4-O-glucosyl-sinapate; 2-O-Trigalloyl-glucose–glucose; and Dicaffeoylshikimic acid), and 2 lipids (LysoPC 18:2; LysoPC 19:0) and three of these metabolites (N-acetyl-L-leucine; Dicaffeoylshikimic acid; and 2-O-trigalloyl-glucose–glucose) presented significant differences between MG and TR stages.

### Amino Acids and Organic Acids

Major contributors to the flavor and nutritional content of fruit include organic acids, amino acids, and saccharides. With a VIP ≥ 1, *value of p* < 0.05, and |Log_2_FC| ≥ 1 as the threshold values for significant difference, a total of 115 flavor-related metabolites with significantly differential expressions in MG and TR were identified, including 45 amino acids and derivatives, 50 organic acids, and 20 saccharides and alcohols (Supplementary Table S4). Moreover, most flavor compounds showed a trend of increase in TR mango fruit and we identified six metabolites among 45 amino acids and derivatives metabolites with a Log2FC ≥ 4, of which four were upregulated and two were downregulated (Table 1). Among the metabolites showing significant increases, Glutathione (GSH), O-phospho-L-serine, γ-glu-cys, and Hexanoyl-L-glycin were increased with fold-changes of 18.78, 12.62, 11.28, and 10.98 expression, respectively. The organic acids (and derivatives), 2-hydroxy-2-methylbutyric acid, tartronate semialdehyde, 4, 5, 6-Trihydroxy-2-oxohexanoic acid, phosphoen olpyruvate, 6-hydroxyhexanoic acid, malonic acid, and DL-glyceraldehyde-3-phosphate were upregulated by 10.13, 11.58, 12.67, 14.58, 14.73, 15.32, and 15.92 times, respectively. Three saccharides and alcohols (3-phospho-D-glyceric acid, D-glucuronic acid, and D-fructose-1,6-biphosphate) were increased with fold-changes of 19.73, 16.39, and 14.78, respectively (Table 1). These differential metabolites may play a key role in the formation of TR fruit taste during fruit ripening.

**Table 1:** Differentially accumulated amino acid, organic acid, and vitamin compounds with VIP ≥ 1, value of *p* < 0.05, and Log_2_FC ≥ 4 (upregulation) or ≤ −4 (downregulation) in TR vs. MG.

**Component name**	**Metabolite name**	**Average content**		**VIP**	**Value of *p***	**Log2FC**	**Type**
		**MG**	**TR**				
Amino acid and derivatives	Glutathione reduced form	n.d.	4.06E+06	1.17E+00	1.89E-03	1.88E+01	up
	O-phospho-L-serine	n.d.	5.68E+04	1.17E+00	4.11E-02	1.26E+01	up
	γ-glu-cys	n.d.	2.25E+04	1.17E+00	3.20E-04	1.13E+01	up
	Hexanoyl-L-glycine	n.d.	1.81E+04	1.17E+00	4.55E-02	1.10E+01	up
	L-arginine	1.35E+07	2.28E+05	1.16E+00	1.46E-02	−5.89E+00	down
	N-α-acetyl-L-ornithine	3.28E+06	3.43E+04	1.17E+00	6.78E-04	-6.58E+00	down
Organic acids and derivatives	DL-glyceraldehyde-3-phosphate	n.d.	5.56E+05	1.17E+00	7.05E-03	1.59E+01	up
	Malonic acid	n.d.	3.67E+05	1.17E+00	8.97E-04	1.53E+01	up
	6-Hydroxyhexanoic acid	n.d.	2.44E+05	1.17E+00	2.62E-02	1.47E+01	up
	Phosphoenolpyruvate	n.d.	2.20E+05	1.17E+00	2.23E-03	1.46E+01	up
	4,5,6-Trihydroxy-2-oxohexanoic acid	n.d.	5.85E+04	1.17E+00	5.78E-03	1.27E+01	up
	Tartronate semialdehyde	n.d.	2.76E+04	1.17E+00	5.54E-03	1.16E+01	up
	2-Hydroxy-2-methylbutyric acid	n.d.	1.01E+04	1.17E+00	5.78E-03	1.01E+01	up
	2-Hydroxyisocaproic acid	8.63E+03	9.81E+05	1.01E+00	1.50E-02	6.83E+00	up
	3-Hydroxybutyric acid	6.82E+04	4.94E+06	1.17E+00	1.23E-02	6.18E+00	up
	2-Hydroxyisobutyric acid	8.17E+03	1.59E+05	1.17E+00	9.27E-03	4.28E+00	up
	4,8-Dihydroxyquinoline-2-carboxylic acid	6.28E+04	n.d.	1.17E+00	1.33E-02	-1.28E+01	down
Saccharides and alcohols	3-Phospho-D-glyceric acid	n.d.	7.84E+06	1.17E+00	2.99E-03	1.97E+01	up
	D-glucuronic acid	n.d.	7.74E+05	1.17E+00	6.20E-03	1.64E+01	up
	D-fructose-1,6-biphosphate	n.d.	2.53E+05	1.17E+00	2.19E-02	1.48E+01	up
	Nystose	3.16E+04	n.d.	1.17E+00	1.40E-02	-1.18E+01	down

n.d., not detectable.

### Metabolomics of Volatiles in Mango Fruits

**Figure 3:**
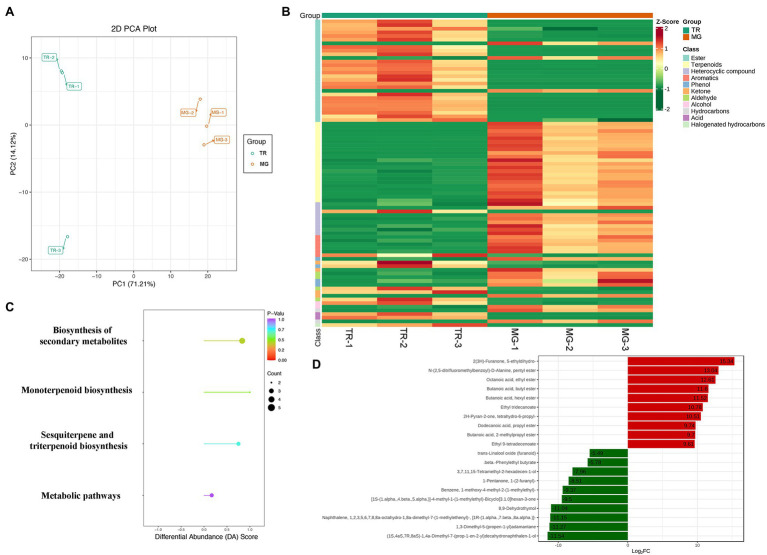
Metabolome analysis of volatile compounds in mango fruits. **(A)** PCA of samples with six biological repetitions at two developmental stages. **(B)** Heat map of different volatile metabolites in MG and TR. **(C)** KEGG pathway enrichment analysis of differential volatile metabolites for TR vs. MG. **(D)** Significantly differentially accumulated volatiles with VIP ≥ 1, *value of p* < 0.01, and |Log_2_FC| ≥ 4.

Plant VOCs are secondary metabolites that play an important role in the flavor of fruits (Coelho et al., 2010). In mango fruits, VOCs mainly consist of esters, alcohols, terpenoids, heterocyclic compounds, aldehydes, and acids. PCA revealed a clear separation of volatile metabolite profiles of the pulps from two varieties, with PC1 and PC2 representing 85.33% of the total components (Figure 3A). With VIP ≥ 1, *value of p* < 0.05, and |Log2FC| ≥ 1 as the threshold values of significant difference, a total of 82 volatile metabolites with significantly differential expressions were identified in “TR vs. MG” (Figure 3B; Supplementary Table S5). Of these, 28 esters and 22 terpenoids act as the main aroma metabolites and constitute the unique aroma of the mango fruit. To screen more precisely for the major volatile metabolites in mango fruit, we narrowed the screening range to *value of p* < 0.01 and |Log2FC| ≥ 4 as thresholds and showed that among the 12 downregulated metabolites, three terpenoids (Naphthalene-octahydro-dimethyl-7-naphthalene, 1,3-dimethyl-5-(propen-1-yl) adamantane, and dimethyl-7-decahydronaphthalen-1-ol) showed more than 10-fold decrease in TR compared to that in MG, Log2FC was −11.15, −11.27, and − 11.54 in expression. In addition, esters occupied six of the seven upregulated metabolites, including octanoic acid, ethyl ester, butanoic acid, butyl ester, butanoic acid, hexyl ester, ethyl tridecanoate, butanoic acid, 2-methylpropyl ester, and propanoic acid, 2-methyl- and octyl ester were upregulated by 12.61, 11.60, 11.52, 10.78, 9.70, and 9.24 times in TR vs. MG, respectively (Table 2). Of these, ethyl-2-methylpropanoate and ethyl butanoate compounds are potentially the most important to mango aroma (Munafo et al., 2016). The KEGG pathway showed biosynthesis of secondary metabolites, sesquiterpene and triterpenoid biosynthesis, and metabolic pathways were the three most significant pathways (Figure 3C). Among the 19 volatile metabolites in Table 2, there were a total of 13 esters and terpenoids, of which six were upregulated and seven were downregulated in expression, and their expression abundance explained the slight difference in mango fruits aroma between MG and TR (Figure 3D).

**Table 2:** Differentially accumulated volatiles with VIP ≥ 1, *p* < 0.01, and Log2FC ≥ 4 (upregulation) or ≤ −4 (downregulation) in TR vs. MG.

**Component name**	**Metabolite name**	**Average content**		**Log2FC**	**VIP**	**Value of *p***	**Type**
		**MG**	**TR**				
Ester	Octanoic acid, ethyl ester	n.d.	5.64E+04	1.26E+01	1.13E+00	7.98E-03	up
	Butanoic acid, butyl ester	n.d.	2.80E+04	1.16E+01	1.13E+00	1.43E-03	up
	Butanoic acid, hexyl ester	n.d.	2.64E+04	1.15E+01	1.13E+00	2.89E-03	up
	Ethyl tridecanoate	n.d.	1.58E+04	1.08E+01	1.13E+00	2.71E-03	up
	Butanoic acid, 2-methylpropyl ester	n.d.	7.48E+03	9.70E+00	1.13E+00	2.59E-03	up
	Propanoic acid, 2-methyl-, octyl ester	n.d.	5.43E+03	9.24E+00	1.13E+00	8.38E-04	up
	β-phenylethyl butyrate	2.30E+06	4.20E+04	−5.78E+00	1.13E+00	8.18E-04	down
Heterocyclic compound	1-Pentanone, 1-(2-furanyl)-	2.02E+05	5.54E+03	−5.18E+00	1.13E+00	2.99E-03	down
	Trans-linalool oxide (furanoid)	2.10E+05	4.69E+03	-5.49E+00	1.13E+00	8.12E-03	down
	Diethylboryl-delta-Valerolactam	3.29E+03	n.d.	−8.51E+00	1.13E+00	3.63E-03	down
Ketone	Acetophenone, 4′-hydroxy-	1.34E+05	3.41E+03	−5.29E+00	1.13E+00	3.66E-03	down
Terpenoids	3-methyl-6-(1-methylethylidene)-Cyclohexene	2.00E+05	8.31E+03	−4.59E+00	1.13E+00	4.35E-03	down
	2-Buten-1-one	5.61E+05	1.41E+04	−5.32E+00	1.13E+00	2.11E-03	down
	4-methyl-1-(1-methylethyl)-Bicyclo hexan-3-one	6.50E+03	n.d.	−9.50E+00	1.13E+00	9.48E-03	down
	Naphthalene-octahydro-dimethyl-7-Naphthalene	2.05E+04	n.d.	−1.12E+01	1.13E+00	6.29E-03	down
	1,3-Dimethyl-5-(propen−1-yl) adamantane	2.22E+04	n.d.	−1.13E+01	1.13E+00	5.10E-03	down
	Dimethyl-7-decahydronaphthalen-1-ol	2.67E+04	n.d.	-1.15E+01	1.13E+00	9.56E-03	down
Acid	Undecylenic Acid	n.d.	3.22E+03	8.48E+00	1.13E+00	9.36E-03	up
Aromatics	1-methoxy-4-methyl-2-Benzene	5.96E+03	n.d.	−9.37E+00	1.13E+00	9.17E-03	down

n.d., not detectable.

### Analysis on Synthesis Pathways of Esters in Mango Fruits

**Figure 4:**
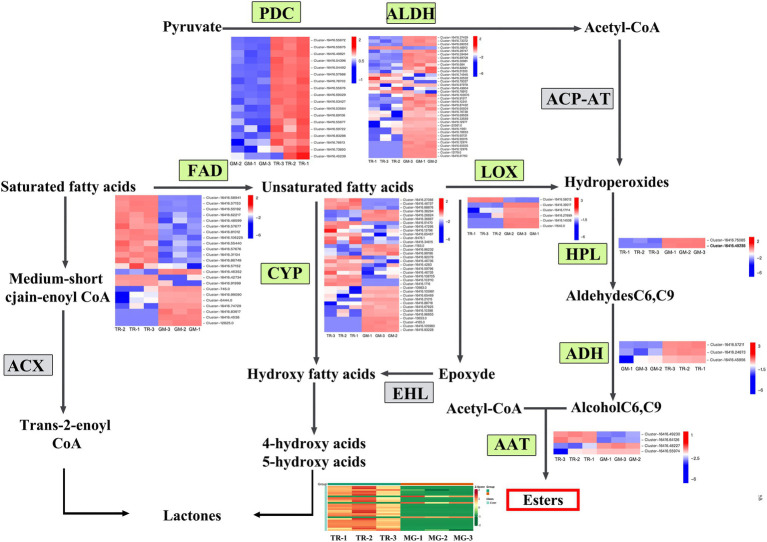
Expression heat map and metabolite content of fatty acid metabolic biosynthesis pathways in mango fruits. Overview of the fatty acid metabolic biosynthesis pathways showing the gene number expansion at each step and their expression profiles in different stages of fruit development. The heat map was drawn using Log2-based FPKM fold values. Red represents high expression, and blue represents low expression.

The volatile organic compounds of plants are secondary metabolites playing an important role in fruit flavor. In mangoes, the volatile organic compounds mainly include esters, terpenes, alcohols, aldehydes, and acids. These compounds showed a declining trend in expression, indicating that the level of many volatile organic compounds declines sharply in MG fruit (Table 2). Among the 82 compounds detected in TR vs. MG (Supplementary Table S5), 50 were esters and 25 showed increases in TR relative to MG. Esters are synthesized mainly through the aliphatic acid pathway. In this study, eight important gene families were identified in the aliphatic acid pathway (Figure 4), namely, acetaldehyde dehydrogenase (ALDH, 36 members), cytochrome P450 (CYP, 33 members), flavin adenine dinucleotide (FAD, 23 members), hydroperoxide lyase (HPL, 2 members), alcohol dehydrogenase (ADH, 3 members), pyruvate decarboxylase (PDC, 18 members), Lipoxygenase (LOX, 6 members), and Arogenate dehydrogenase (AAT, 4 members; Supplementary Table S6). According to the expression analysis, it was found that the expression levels of FAD, HPL, and ADH decreased with fruit development, which is consistent with the decreasing trend of ester metabolite content. Among them, ADH is involved in the biosynthesis pathway of aroma volatiles in fruits by converting aldehydes to alcohols and providing substrates for ester formation (Manríquez et al., 2006). ALDH (30 and 6 genes showing significant up- and downregulation, respectively) and CYP (18 and 17 genes significantly up- or downregulated, respectively) indicating a general trend for gene upregulation than downregulation. CYP genes encode an enzyme that can catalyze a wide range of reactions but has been identified as responsible for the C10 hydroxylation of α-pinene to myrtenol, leading to different flavors in strawberry fruits (Aharoni et al., 2019). The differential expressions observed here in CYP family gene members may similarly impact on mango flavors in the transition from MG to the TR stage of fruit ripening. In addition, the other ester-related gene families of PDC, LOX, and AAT also showed differential regulations, suggesting that esters are important components of mango flavor substances.

### Analysis of Carotenoid Biosynthesis

**Figure 5:**
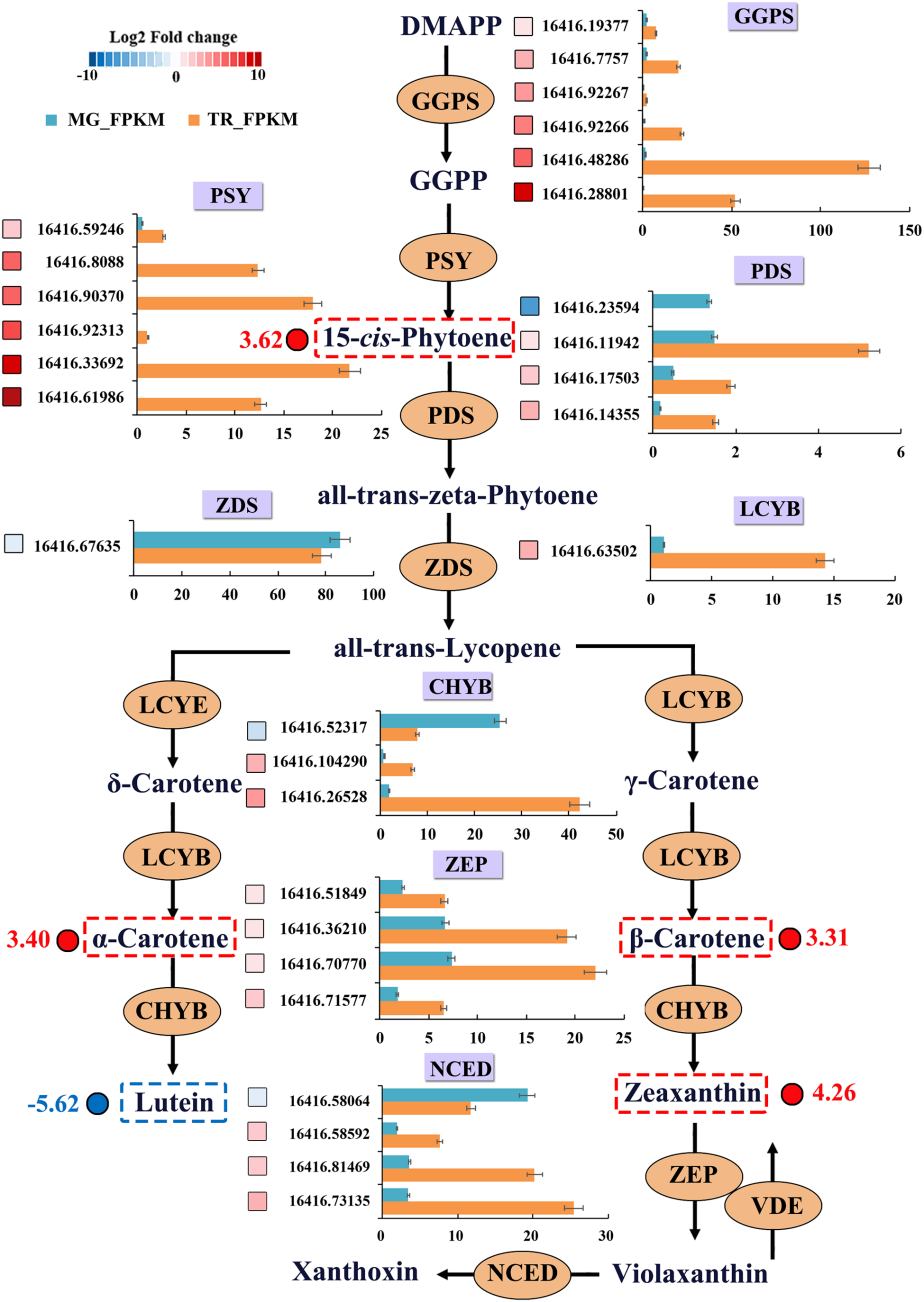
DEGs and differential metabolites of carotenoid-biosynthesis pathway in mangoes. DEGs were recruited by |Log_2_FC| ≥ 1. Blue and red boxes indicate downregulated and upregulated transcripts, respectively. Grids with 10 different gray-scale levels show the Log_2_FC value, with the Log_2_FC values 0–1, 1–2, 2–3, 3–4, 4–5, 5–6, 6–7, 7–8, 8–9, and 9–10 represented by gray-scale levels 1–10, respectively. *X*-axis represents the transcript value of FPKM. Expression patterns of differential metabolites are indicated at the side of each metabolite.

Carotenoids are crucial players in photosynthesis and lipid peroxidation in plants and their content impacts on the color and appearance of fruits (Chisté et al., 2014). The dynamic changes in gene expression and differential metabolite levels in the carotenoid synthesis pathway in TR vs. MG were analyzed for a better understanding of the molecular genetics of mango pulp coloration (Figure 5; Table 3). The results of the analysis of significant DEGs indicated that 26 could be associated with changes in the carotenoid metabolome (Figure 5). Among them, the GGPS participating in carotenoid precursor synthesis showed a significant increasing trend in expression from MG to the TR stage. The 15-cis PSY genes playing an important role in the synthesis of β-carotene precursors all showed a similarly significantly increasing trend in expression. Among the PDS genes, three showed upregulated expression and one had a downregulated expression. The expression level of gene Cluster-16416.67635 encoding ZDS was significantly increased by 1.21 times in TR fruit. The expression level of LCYB gene, the key enzyme in carotene synthesis, was significantly upregulated by 3.26 times, indicating a rapid increase of β-carotene synthesis in TR. The results of carotenoid metabolome assays showed that the content of β-carotene was significantly increased by 3.31 times in TR vs. MG, suggesting that the high expression of LCYB gene can cause carotenoid accumulation. MG showed a faint yellow color which transitioned to orange in the ripening process and this can be attributed to the differences in the levels of α- and β-carotenes. CHYBs catalyze the synthesis of zeaxanthin from β-carotene or lutein from α-carotene and thus prevent the accumulation of carotenes. Among the CHYB genes, two were upregulated and one downregulated in MG fruit which might explain the enhanced accumulation of carotenes at the TR stage of ripening. Similarly, the upregulation in MG of all ZEP genes and three out of four NCED genes that operate down-stream of CHYBs in the β-carotene branch of carotenoid synthesis might also have contributed to relative decreased pool of β-carotene at the MG stage.

**Table 3:** Differentially accumulated carotenoid compounds with *p* < 0.05 and Log2FC ≥ 1 (upregulation) or ≤ −1 (downregulation) in TR vs. MG.

**Compound**	**Value of *p***	**Fold change**	**Log2FC**	**Type**
Violaxanthin-myristate-laurate	N/A	20.17544	4.334528	up
Zeaxanthin	0.058697824	19.09868	4.255401	up
β-cryptoxanthin myristate	N/A	14.36945	3.844933	up
Zeaxanthin palmitate	0.047466738	13.24232	3.727084	up
(E/Z)-phytoene	0.028656479	12.25841	3.615701	up
α-carotene	0.03099068	10.53305	3.396851	up
5,6epoxy-lutein-caprate-palmitate	0.034620512	10.05489	3.329826	up
β-carotene	0.031209197	9.891864	3.306242	up
Lutein oleate	0.034803712	8.762542	3.131349	up
β-cryptoxanthin	0.035113107	7.413661	2.890186	up
Violaxanthin dipalmitate	0.057827363	5.674468	2.504485	up
Violaxanthin-myristate-palmitate	0.060803051	4.280488	2.097775	up
Violaxanthin myristate	0.051086948	4.214062	2.075211	up
Zeaxanthin dimyristate	N/A	2.157895	1.109624	up
Lutein	0.002168091	0.020309	−5.62171	down

### Analysis of Transcription Factors and RT-qPCR Validation

TFs are key players in controlling the expression of structural genes in secondary metabolite biosynthesis. In this study, 563 DEGs in TR vs. MG were found to be TFs. In “TR vs. MG,” the expression of 439 TFs was upregulated, while 124 TFs showed downregulation. TFs were annotated as NAC, WRKY, bHLH, MYB, AP2/ERF, bZIP, HSF, COP1, or MADS-box (Supplementary Table S7). The bZIP TF, HY5 (16416.43081), was downregulated by 1.82 times in TR vs. MG. In addition, 76 of important MYBs were selected from “TR vs. MG.” among which 56 genes were upregulated and 20 genes were downregulated in expression. 16416.57250, 16416.71538, and 16416.47912 were reduced by 8.90, 8.61, and 8.42 times, respectively (Supplementary Table S7). As vital regulatory TFs in carotenoid-biosynthesis pathways, bHLHs are involved in the regulation of important carotenoid-biosynthesis genes, such as LCYB. In “TR vs. MG,” 96 differentially expressed bHLHs were detected, 89 of them were upregulated, and 7 were downregulated in expression. NACs, the largest TF family in plants, are expressed in different tissues and at various stages of plant development. These participate in the regulation in plant growth and development and the plant responses to environmental stresses. In this study, 116 differentially expressed NACs were detected, with 73 showing upregulation and 43 with downregulated expression. 16416.58202 was annotated as NAC083. Its expression in TR was the highest, up to 165.27, showing a significant increase of 4.06 times compared with that in MG. Some studies have shown that CrMYB68 can directly interact with CrBCH2 and CrNCED5 leading to carotenoid accumulation (Zhu et al., 2020). CpbHLH1 and CpbHLH2 individually regulate the transcription of lycopene β-cyclase genes (CpCYC-B and CpLCY-B) during papaya fruit ripening (Zhou et al., 2019).

To validate the key results of the RNA-Seq, in this study, 13 carotenoid-biosynthesis pathway genes, 3 MYB, 2 bHLHs, and 2 NAC family TFs were selected, and their expression levels in MG and TR samples were analyzed using RT-qPCR (Supplementary Figure S3). The expression levels of these structural genes were in line with those of the RNA-Seq results.

## Discussion

### Effects of Sugars, Organic Acids, and Amino Acids on the Flavor of Mango Fruit

The fruit flavor quality is mainly influenced by saccharides and organic acids involved in carbohydrate metabolism and the flavor of mangoes may be largely determined by the content and type of these different metabolites. The flavor of fruits is generally determined by the ratio and concentration of volatile compounds, such as phenols and alcohols, as well as sugars, organic acids, and amino acids. As for the changes in flavor that occur during fruit ripening, a few specific metabolites have been focused on including saccharides (fructose, glucose, and sucrose), organic acids, amino acids, and alcohols (Chen et al., 2009; Lombardo et al., 2011). Up to now, there are no studies on the effects of secondary metabolites on mango fruit flavor. In order to gain insight into metabolites contributing to mango flavor, broad-target metabolomics by HPLC-MS/MS was used to characterize the changes in metabolite levels that occur during the transition between the MG and TR stages of fruit development. A total of 603 related metabolites, such as sugars and organic acids, were identified, of which 309 metabolites were accumulated to a higher degree in MG mango fruit (Supplementary Table S8). A great deal of carbohydrates (including 20 saccharides) was identified and analyzed in mango pulps (Supplementary Table S4). Among these 20 saccharides, 14 saccharides exhibited increased concentrations at the TR stage. Among them, five increased saccharides (D-Glucoronic acid, D-Fructose-1,6-biphosphate, D-Ribose, Mannitol*, and Dulcitol*) were the main saccharides contributors of flavor in mango fruits. In addition, a total of 50 organic acids differentially expressed were identified, of which 34 organic acids showed significantly increased concentrations at the TR stage, partly contributing to the changes in fruit flavor. The constitution and richness of amino acids are key indexes of nutrition quality and are also important to the flavor (Choi et al., 2012). Among the 73 amino acids identified in this study, 45 displayed differential accumulations in TR vs. MG (Table 1) and four amino acids (Glutathione reduced form, O-phospho-L-serine, γ-glu-cys, and Hexanoyl-L-glycine) were increased in TR (Table 1), with a Log_2_FC > 10. These results suggest that the differences in constitution and richness of amino acids also affect fruit flavor.

### Esters and Terpenoids May Be the Main Volatiles Constituting the Aroma of Mango

There were 143 volatile-related metabolites identified in this study, of which 82 volatile metabolites showed differential accumulation between MG and TR stages of mango pulp development (Supplementary Table S9). A large number of ester and terpenoid compounds were identified and their levels analyzed in this study. Pino and Mesa (2010) reported that ethyl-2-methylpropionate, ethyl butyrate, and methyl benzoate were the main esters determining the aroma of mango. Esters and terpenes are usually produced during fruit ripening by lipid metabolism; however, lipid metabolism is further enhanced by ripening temperature (Lalel et al., 2003). Esters are mainly synthesized by the fatty acid pathway, and eight important gene families were identified in this pathway (Figure 4). It was found that PDC, ALDH, FAD, and CYP gene families had large numbers of members: 18, 36, 23, and 35, respectively. These genes, especially ALDH genes, may play crucial roles in ester metabolism. Terpenoids have been reported to be the main fruit aroma substances, with terpinene having floral and sweet aroma properties, while γ-Terpinene and myrcene are responsible for the aroma of green mango fruits (Engel and Tressl, 1983; Tamura et al., 2001). Terpinene is the main volatile compound in cv. Willard and Parrot mango fruits (MacLeod and Pieris, 1984). In our study, γ-Terpinene was present as the main terpenoid in the “Golden Phoenix” mango fruit. Changes in the abundance of volatiles during ripening can determine the commercial value of mango fruits (Thiruchelvam et al., 2020). The type and concentration of volatile compounds vary considerably among cultivars. Interestingly, in our study, 22 terpenoids were upregulated in MG mango fruit. These differences explain the reason why MG fruits have a fruity aroma.

### Flavone/Isoflavone and Carotenoids Biosynthesis in Mango Fruits

Mango fruit color is mainly composed from the flavonoids in vacuoles and the plastidial carotenoids and chlorophyll in pulps. In addition, another important factor for attractive mangoes is their fruit peel color which is determined by their contents of carotenoids, chlorophyll, and flavonoids (Karanjalker et al., 2018). There are more than 600 types of known carotenoids, which provide pigmentation to many plant organs, including fruits, and are vital players in consumer appreciation and economic values of plant products. In the ripening stage of apples, the background color is composed of chlorophyll, carotenoids, and flavonoids. The content and proportion of these three pigments determine the background color of the pericarps, thereby affecting the surface color (Mark et al., 2010). During mango ripening, its chlorophyll is almost completely degraded at 22°C. Anthocyanin contents also decrease slightly, while levels of carotenoids, mainly β-carotene and violaxanthin, are increased (Karanjalker et al., 2018). Furthermore, flavonoids are important pigments, which can produce a series of color from yellow to red and from faint yellow to orange (Ni et al., 2020). The transcriptomic analyses performed in this study identified some changes in the biosynthesis of flavonoid metabolite biosynthetic pathways between MG and TR fruit stages. From the metabolomics data, KEGG enrichment analysis also indicated that the concentration of metabolites in two phenolic pathways (“flavonoid biosynthesis” and “flavone/isoflavone biosynthesis”) in pulps was significantly different in fruits pulps at the MG and TR stages. It was confirmed through analysis of flavonoid metabolite profiles that the accumulation of flavonols, flavonoid, and isoflavone metabolites was increased in TR (Supplementary Table S4). In the biosynthesis pathway of phenylpropanoids, the expression levels of 4CL, C4H, CHI, and histohaematin P450 (FNS/IFS) genes presented positive correlations with the accumulation of flavonoids and isoflavones. Previous studies have shown that the structural genes in the phenylpropanoid pathway determine the accumulation of flavonoids and that these are partly regulated by MYB TFs (Supplementary Table S7). These, together with bHLH3 and WD40, can form MBW complexes, jointly leading to the biosynthesis of phenylpropanoids and proanthocyanin (PA; Nemie-Feyissa et al., 2015). In this study, many DEGs between MG and TR for MYB and bHLH TFs were detected, which may affect flavone/isoflavone biosynthesis during fruit ripening. However, their roles in this process require further experimental verification.

## Data Availability Statement

The original contributions presented in the study are publicly available. This data can be found at: https://www.ncbi.nlm.nih.gov/bioproject/, National Center for Biotechnology Information (NCBI) BioProject database under accession number PRJNA751842.

## Author Contributions

ZW and LP designed the experiments. WG, ML, and DH conducted the experiments and analyzed the results. ZW, MS, and WG prepared the manuscript. All authors have read and approved the manuscript for publication.

## Funding

This work was supported by the YEFICRC Project of Yunnan Province Key programs (2019ZG00907) and Science Research Fund Project of Yunnan Provincial Department of Education (2020J0247) and Yunnan Fundamental Research Projects (202101AU070094).

## Conflict of Interest

The authors declare that they have no known competing financial interests or personal relationships that could have appeared to influence the work reported in this paper.

## Publisher’s Note

All claims expressed in this article are solely those of the authors and do not necessarily represent those of their affiliated organizations, or those of the publisher, the editors and the reviewers. Any product that may be evaluated in this article, or claim that may be made by its manufacturer, is not guaranteed or endorsed by the publisher.

## Footnotes

^1^ http://www.anpel.com.cn/

^2^ www.shimadzu.com.cn/

^3^ www.appliedbiosystems.com.cn/

^4^ https://sciex.com.cn/

^6^ http://itak.feilab.net/cgi-bin/itak/index.cgi

^7^ http://www.geneontology.org/

^8^ https://www.rproject.org/

## Abbreviations

4CL: 4-Coumaric acid coenzyme A ligase
ABA: Abscisic acid
bHLH: Basic helix–loop–helix
bZIP: Basic leucine zipper
C4H: Cinnamic acid 4-hydroxylase
CHI: Chalcone isomerase
COG: Clusters of orthologous groups of proteins database
DEG: Differentially expressed gene
FPKM: Fragments per kilobase of exon model per million mapped reads
GGPS: Geranylgeranyl diphosphate synthase
GO: Gene Ontology
IPI: Gentian isoprene pyrophosphate isomerase
KEGG: Kyoto encyclopedia of genes and genomes
LCYB: Lycopene-β-cyclase
LycE: Lycopene-epsilon-cyclase
MYB: v-myb avian myeloblastosis viral oncogene homolo
PA: Procyanidins
PAL: Phenylalanine ammonia-lyase
PDS: Phytoene desaturase
PSY: Phytoene synthase
TFs: Transcription factors
ZDS: Zeta-carotene desaturase.
