# Supplementary material for: Integrated Metabolome and Transcriptome Analysis of Fruit Flavor and Carotenoids Biosynthesis Differences Between Mature-Green and Tree-Ripe of cv. “Golden Phoenix” Mangoes (Mangifera indica L.)
Source: Front Plant Sci. 2022 Feb 24;13:816492. doi: 10.3389/fpls.2022.816492 (PMC8907839; doi:10.3389/fpls.2022.816492)
Supplement: Supplementary file 1 [file Data_Sheet_1.PDF]

Integrated Metabolome and Transcriptome Analysis of Fruit Flavor and Carotenoids Biosynthesis Differences Between Mature-green and Tree-ripe of cv. 'Golden Phoenix' Mangoes (*Mangifera indica* L.)

Lei Peng[1†], Wenke Gao[1†], Miaoyu Song[2†], Minghai Li[1], Dinan He[1], Ziran Wang[1*]

[a] College of Horticulture and Landscape, Yunnan Agricultural University, Kunming, 650224, PR China

[b] College of Horticulture, China Agricultural University, Beijing, 100193, PR China

Lei Peng: penglei169@126.com

Wenke Gao: 1098752607@qq.com

Miaoyu Song: songmiaoyu@cau.edu.cn

Minghai Li: 3192483171@qq.com

Dinan He: 775488733@qq.com

Ziran Wang: wangziran@cau.edu.cn

*Correspondence:

Ziran Wang: wangziran@cau.edu.cn

Running title:

Metabolomic and Transcriptomic analysis of Mango fruit flavor and color

Table S1. The fruit quality of mature-green and tree-ripe cv. "Golden Phoenix" mango fruits

**Types**	**Length (mm)**	**Fresh weight (g)**	**Soluble solids content (◦Brix)**	**Titrate acid (%)**	**Firmness (N/cm^2)**
**MG**	135.55 ± 0.42aA	296.40 ± 0.11aA	13.36 ± 0.70bA	13.26 ± 5.15aA	9.64 ±0.36aA
**TR**	132.50 ± 0.78aA	307.21 ± 0.14aA	16.72 ± 3.57aA	9.96±3.39aA	7.76 ± 0.33aA

Table S2. Primer sequences of genes used for verification of digital gene-expression results by RT-qPCR

Gene name	Seq ID	Forward primer (5'→3')	Reverse primer (5'→3')
Actin	Reference gene	GCCATTCAAGCCGTGCTTT	TGGGAACAGTGTGGCTGACA
MiGGPS-1	c28801	CCTTCGTGTGATTGCTGAGA	CTGCACCAGCCAATAATCCT
MiGGPS-2	c48286	TCCTTCGTGTGATTGCTGAG	CTGCACCAGCCAATAATCCT
MiPSY-1	c33692	TACTCCGGGATGTTGGAGAG	CCCATACCGGCCATCTACTA
MiPSY-2	c8088	GGCCTAACGCTTCACACATT	GGTCCATCCTCATTCCTTCA
MiPSD-1	c11942	CACTGACACAGCCTGGAGAA	TCTGGTGAGCAAGAACGATG
MiPSD-2	c23594	TTGAGCTTGTGTGGAAGTGC	AGCAGAAGCACGAAAGGAAG
MiZDS	c67635	AGAATGTGGGATCCTGTTGC	CTTTCTAATGGGGCCACTCA
MiLCYB	c63502	CCAGGAAAGAATGGTGGCTA	ACAATAGGGGCTGCTGCTAA
MiCHYB-1	c52317	CGTTCCAGCCATAGCTCTTC	ATAAGGTACGTTGGCGATGG
MiCHYB-2	c26528	TGTTTGGCACATTTGCTCTC	GAAGAGCTATGGCTGGAACG
MiZEP	c70770	AACCACCTCAACCGACTTTG	ACCAACAGGCTCCCTTCTTT
MiNCED-1	c58064	CGGAGAACCCTGTCTCTCAC	AACGAAACGGTTGGTTTCAG
MiNCED-2	c73135	TTTCCCTAAAGCCATTGGTG	GGAAGGAGTGATGCGAACAT
MiMYB-1	c50464	CGGAGGATGATAACGACGAT	TTTCAACAAACGTCCCAACA
MiMYB-2	c57250	GTTTGGTGCTCAAGCTCTCC	CAATTTGAAACCTCCGCAAT

MiMYB-3	c78790	GAGTTCGTTGGCTCTTCTGG	GGCAGTGCCATTATTTGTCC
MibHLH-1	c91351	AACTGGGAAATTGCGTTCAC	TTCTAACCCTTTCGGCAATG
MibHLH-2	c19213	TCACTCAGCATGCCAAGAAG	AATCTGTCCAACTGCCATCC
MiNAC-1	c49791	TCCGTTTTCATCCCACTGAT	GTGGCCTTCCAATAACCAGA
MiNAC-2	c36084	GGGTTCTCTTGTGGGATTGA	CCGGTGGACACGTTTACTTT

Table S3. Summary of the sequencing assembly

Sample	Raw Reads	Clean Reads	Mapped Read	Mapped rate (%)	Clean Base (G)	Q20 (%)	Q30 (%)	GC Content (%)
MG-1	50507026	47074934	42274700	89.80	7.06	98.61	95.82	43.75
MG-2	47023848	45179460	40139580	88.84	6.78	98.52	95.53	43.7
MG-3	46537760	44645604	39493010	88.46	6.7	98.55	95.69	43.72
TR-1	47985896	46246564	39088254	84.52	6.94	98.58	95.78	44.03
TR-2	44686908	42775682	36315182	84.90	6.42	98.67	95.84	43.99
TR-3	45375274	43384378	36867724	84.98	6.51	98.73	95.97	43.99

Table S4. The classification of compounds detected by the metabolome in TR *vs.* MG.

Index	Compounds	MG	TR	VIP	Log2FC	Type
**Alkaloids**						
mws0146	Nicotinic Acid Methyl Ester (Methyl Nicotinate)	n.d.	8.42E+06	1.17E+00	1.98E+01	up
mws0983	N-Oleoylethanolamine	4.53E+04	4.14E+05	1.16E+00	3.19E+00	up
mws0704	O-Phosphorylethanolamine	5.67E+04	2.14E+05	1.15E+00	1.92E+00	up
mws2171	Camalexin	2.22E+03	7.89E+03	1.16E+00	1.83E+00	up
pmb0484	Choline	9.72E+06	2.84E+07	1.16E+00	1.55E+00	up
pmp001198	6-Deoxyfagomine	2.45E+06	6.40E+06	1.15E+00	1.38E+00	up
pmb0782	Piperidine	2.53E+06	6.10E+06	1.15E+00	1.27E+00	up
pmb2211	Cocamidopropyl betaine	3.73E+04	1.47E+04	1.06E+00	-1.34E+00	down
HJKP000649	N-benzylformamide	3.43E+06	7.35E+05	1.16E+00	-2.22E+00	down
Hmtp000776	4,5,6-Trihydroxy-2-cyclohexen-1-ylideneacetonitrile	7.75E+05	1.53E+05	1.16E+00	-2.35E+00	down
pmp001287	N-Benzylmethylene isomethylamine	1.84E+07	3.41E+06	1.16E+00	-2.44E+00	down
pme1691	Acetylcholine	6.22E+06	4.65E+05	1.17E+00	-3.74E+00	down
pme2268	Trigonelline	3.89E+06	1.70E+05	1.17E+00	-4.52E+00	down
Zmtn001624	N-Acetylisatin	5.17E+04	n.d.	1.17E+00	-1.25E+01	down
pme2292	Putrescine	1.68E+06	n.d.	1.17E+00	-1.75E+01	down

**Amino acids and derivatives**						
pme1086	Glutathione reduced form	n.d.	4.06E+06	1.17E+00	1.88E+01	up
pme2602	O-Phospho-L-serine	n.d.	5.68E+04	1.17E+00	1.26E+01	up
pme2563	γ-Glu-Cys	n.d.	2.25E+04	1.17E+00	1.13E+01	up
pme2853	Hexanoyl-L-glycine	n.d.	1.81E+04	1.17E+00	1.10E+01	up
Lmrj002087	L-Isoleucyl-L-Aspartate	1.10E+04	1.59E+05	1.14E+00	3.85E+00	up
pme1286	S-(5'-Adenosy)-L-homocysteine	5.47E+03	7.87E+04	1.17E+00	3.85E+00	up
mws4134	Oxiglutatione	1.28E+04	1.49E+05	1.15E+00	3.54E+00	up
pme3382	N-Acetyl-L-threonine	6.58E+03	6.28E+04	1.15E+00	3.25E+00	up
Lmbp000123	L-Homomethionine	2.82E+05	2.56E+06	1.17E+00	3.19E+00	up
pme3033	N, N-Dimethylglycine	4.10E+05	2.07E+06	1.17E+00	2.34E+00	up
Lmrj001698	L-Seryl-L-Isoleucine	6.54E+03	3.05E+04	1.15E+00	2.22E+00	up
pme0253	N-Acetyl-L-leucine	3.52E+03	1.57E+04	1.14E+00	2.15E+00	up
pme1987	L-Alanine	2.83E+03	1.23E+04	1.16E+00	2.12E+00	up
mws0124	N-(3-Indolylacetyl)-L-alanine	1.89E+04	7.27E+04	1.16E+00	1.94E+00	up
pme1419	L-Methionine methyl ester	3.14E+04	1.14E+05	1.17E+00	1.87E+00	up
pme3017	2-Aminoisobutyric acid	1.09E+06	3.76E+06	1.16E+00	1.79E+00	up
Lmhp001670	L-Valyl-L-Leucine	2.96E+04	9.37E+04	1.13E+00	1.66E+00	up
mws0227	L-Leucine*	4.02E+05	1.21E+06	1.16E+00	1.59E+00	up

Lmhp002001	L-Valyl-L-Phenylalanine	1.78E+04	5.28E+04	1.10E+00	1.56E+00	up
mws0875	L-Cysteinyl-L-glycine	1.47E+05	4.01E+05	1.15E+00	1.45E+00	up
pme0010	L-Serine	1.53E+05	3.71E+05	1.11E+00	1.28E+00	up
mws0520	N-Acetyl-L-tyrosine	5.77E+03	1.38E+04	1.13E+00	1.26E+00	up
mws5041	L-Glycyl-L-isoleucine*	2.57E+04	5.78E+04	1.14E+00	1.17E+00	up
ML10198895	N-Methylglycine	1.30E+04	2.89E+04	1.12E+00	1.16E+00	up
mws0736	N-Glycyl-L-leucine*	3.53E+04	7.71E+04	1.11E+00	1.13E+00	up
mws0258	L-Isoleucine*	8.04E+05	1.70E+06	1.12E+00	1.08E+00	up
pmb0962	L-Lysine-Butanoic Acid	2.28E+04	4.78E+04	1.03E+00	1.07E+00	up
pme0026	L-Lysine	1.16E+07	4.80E+06	1.16E+00	-1.27E+00	down
ML10181668	Cycloleucine*	7.12E+05	2.91E+05	1.13E+00	-1.29E+00	down
pme0193	L-Glutamine	1.23E+07	4.96E+06	1.15E+00	-1.32E+00	down
Lmrj002793	Cyclo(Phe-Glu)	3.80E+04	1.46E+04	1.13E+00	-1.38E+00	down
pme0075	N-Acetyl-L-glutamic acid	1.00E+05	3.49E+04	1.14E+00	-1.52E+00	down
mws0250	L-Tyrosine	5.77E+07	1.78E+07	1.16E+00	-1.70E+00	down
pmb0464	L-Aspartic acid-O-diglucoside	6.10E+06	1.88E+06	1.15E+00	-1.70E+00	down
mws0219	L-Aspartic Acid	8.71E+06	2.54E+06	1.16E+00	-1.78E+00	down
pmb2857	L-Glutamic acid-O-glycoside	4.96E+04	1.43E+04	1.10E+00	-1.79E+00	down
mws0582	S-(Methyl)glutathione	8.48E+05	2.21E+05	1.16E+00	-1.94E+00	down

pme0021	L-Phenylalanine	3.53E+07	6.96E+06	1.16E+00	-2.35E+00	down
pme0006	L-Proline	5.37E+07	8.37E+06	1.17E+00	-2.68E+00	down
pme0008	L-Citrulline	5.65E+05	8.73E+04	1.13E+00	-2.69E+00	down
pme2527	L-Ornithine	4.95E+04	7.22E+03	1.14E+00	-2.78E+00	down
pme2735	S-Adenosyl-L-methionine	8.39E+05	6.14E+04	1.16E+00	-3.77E+00	down
mws0629	L-Aspartyl-L-Phenylalanine	7.25E+04	4.62E+03	1.16E+00	-3.97E+00	down
mws0260	L-Arginine	1.35E+07	2.28E+05	1.16E+00	-5.89E+00	down
Zmyn000155	N-α-Acetyl-L-ornithine	3.28E+06	3.43E+04	1.17E+00	-6.58E+00	down
**Flavonoids**						
Lmmp003767	Kaempferol-7-O-glucuronide	n.d.	3.41E+03	1.17E+00	8.56E+00	up
Zmdp005767	Formononetin-7-O-(6"-Malonyl) glucoside	2.05E+03	1.78E+04	1.17E+00	3.12E+00	up
mws0032	Myricetin	2.60E+03	1.36E+04	1.16E+00	2.39E+00	up
Lmmp003271	Gossypetin-8-O-glucoside	7.06E+03	2.50E+04	1.13E+00	1.83E+00	up
mws2209	Kaempferol-3-O-glucoside (Astragalin)*	6.23E+06	1.76E+07	1.16E+00	1.50E+00	up
pme2459	Luteolin-7-O-glucoside (Cynaroside)	6.05E+06	1.70E+07	1.16E+00	1.49E+00	up
Lmyp003139	Quercetin-3-O-(6"-galloyl) galactoside	1.81E+04	n.d.	1.17E+00	-1.10E+01	down
**Lignans and Coumarins**						
mws1014	Fraxetin (7,8-Dihydroxy-6-methoxycoumarin)	n.d.	1.96E+05	1.17E+00	1.44E+01	up
HJN083	Lariciresinol-4'-O-glucoside	n.d.	6.16E+04	1.17E+00	1.27E+01	up

Cmpp003619	6,7-Dihydroxy-4-methylcoumarin	3.33E+04	1.85E+05	1.16E+00	2.47E+00	up
Lmtn003096	Secoisolariciresinol 4-O-glucoside	6.64E+04	2.12E+05	1.13E+00	1.68E+00	up
Lmqn001932	Syringaresinol-4'-O-glucoside	1.60E+04	4.00E+04	1.12E+00	1.32E+00	up
pme2993	Scopoletin (7-Hydroxy-5-methoxycoumarin)	1.92E+04	4.23E+04	1.14E+00	1.14E+00	up
Rfmb26201	Syringaresinol-4'-O-(6"-acetyl) glucoside	6.04E+04	1.29E+05	1.14E+00	1.09E+00	up
mws0097	Pinoresinol*	9.41E+04	4.68E+04	1.10E+00	-1.01E+00	down
Lmdn004267	Epipinoresinol*	9.52E+04	4.69E+04	1.09E+00	-1.02E+00	down
pmn001663	Syringaresinol	1.78E+05	1.55E+04	1.16E+00	-3.52E+00	down
**Lipids**						
pmp001281	LysoPC 18:1	n.d.	3.69E+06	1.17E+00	1.86E+01	up
pma1303	LysoPC 16:2	n.d.	2.94E+05	1.17E+00	1.50E+01	up
pmb0862	LysoPC 12:0	n.d.	7.04E+04	1.17E+00	1.29E+01	up
Lmhp007836	LysoPE 16:3	n.d.	2.72E+04	1.17E+00	1.16E+01	up
Lmhp009769	LysoPE 17:1	n.d.	1.83E+04	1.17E+00	1.10E+01	up
pmd0136	LysoPC 18:0(2n isomer)	n.d.	1.38E+04	1.17E+00	1.06E+01	up
Lmhp007840	LysoPC 19:2	n.d.	1.09E+04	1.17E+00	1.02E+01	up
Lmhp008337	LysoPE 14:0(2n isomer)	7.68E+02	4.10E+04	1.16E+00	5.74E+00	up
pmb0864	LysoPE 14:0	1.68E+04	7.54E+05	1.17E+00	5.49E+00	up
pmb0863	LysoPC 16:2(2n isomer)	9.80E+03	2.53E+05	1.17E+00	4.69E+00	up

Lmhp009034	LysoPE 16:1	8.14E+04	1.91E+06	1.16E+00	4.55E+00	up
pmd0130	LysoPC 14:0	6.99E+03	8.58E+04	1.16E+00	3.62E+00	up
pmp001270	LysoPC 16:1	2.89E+05	3.49E+06	1.17E+00	3.59E+00	up
mws0126	LysoPC 18:0	1.32E+04	1.27E+05	1.17E+00	3.26E+00	up
pmb0883	LysoPE 18:0	1.41E+03	1.25E+04	1.15E+00	3.15E+00	up
mws0289	LysoPE 18:1	1.82E+05	1.55E+06	1.17E+00	3.09E+00	up
mws2623	11-Octadecanoic acid (Vaccenic acid)	1.66E+06	1.32E+07	1.17E+00	2.99E+00	up
Lmhp011388	2-α-Linolenoyl-glycerol	8.46E+04	6.11E+05	1.17E+00	2.85E+00	up
pmd0160	LysoPE 16:0(2n isomer)	1.24E+05	7.55E+05	1.17E+00	2.60E+00	up
mws0396	Elaidic Acid	6.14E+06	3.58E+07	1.16E+00	2.54E+00	up
Hmsn000210	Methyl 7,10-hexadecadienoate	1.23E+06	6.05E+06	1.17E+00	2.30E+00	up
pmb0855	LysoPC 16:0	4.49E+06	2.08E+07	1.17E+00	2.21E+00	up
pmb0876	LysoPE 16:0	2.80E+06	1.28E+07	1.17E+00	2.19E+00	up
pmp001273	LysoPC 18:2	2.37E+05	9.17E+05	1.16E+00	1.95E+00	up
Lmhp011562	1-α-Linolenoyl-glycerol	1.41E+05	5.18E+05	1.16E+00	1.88E+00	up
Lmbn005923	Crepenynic acid	2.63E+04	8.46E+04	1.15E+00	1.69E+00	up
Lmhp008744	1-α-Linolenoyl-glycerol-2,3-di-O-glucoside	2.90E+03	9.20E+03	1.07E+00	1.67E+00	up
Lmhp009773	1-α-Linolenoyl-glycerol-3-O-glucoside	3.31E+03	1.04E+04	1.12E+00	1.64E+00	up
Lmhp008763	LysoPE 16:1(2n isomer)	5.57E+05	1.67E+06	1.16E+00	1.59E+00	up

mws0366	γ-Linolenic Acid*	1.15E+07	3.39E+07	1.16E+00	1.56E+00	up
pmb0854	LysoPC 18:3	4.55E+05	1.32E+06	1.15E+00	1.54E+00	up
mws0367	α-Linolenic Acid*	1.16E+07	3.35E+07	1.16E+00	1.53E+00	up
mws0383	10-Heptadecenoic Acid	2.21E+05	6.21E+05	1.14E+00	1.49E+00	up
Lmhp008513	2-α-Linolenoyl-glycerol-1,3-di-O-glucoside	2.86E+03	7.75E+03	1.06E+00	1.44E+00	up
Lmhp008801	LysoPE 18:3	2.38E+05	6.05E+05	1.14E+00	1.35E+00	up
pmd0132	LysoPC 16:0(2n isomer)	3.85E+05	9.64E+05	1.16E+00	1.32E+00	up
Lmhp009526	2-α-Linolenoyl-glycerol-1-O-glucoside	3.07E+03	7.48E+03	1.15E+00	1.28E+00	up
mws0361	Palmitoleic Acid	8.07E+03	1.91E+04	1.13E+00	1.24E+00	up
pmn001610	Eicosadienoic acid	1.59E+04	3.71E+04	1.16E+00	1.22E+00	up
pmb2228	LysoPC 19:0	3.15E+03	6.89E+03	1.07E+00	1.13E+00	up
Lmhp010334	2-Linoleoylglycerol-1-O-glucoside	2.53E+03	5.45E+03	1.14E+00	1.11E+00	up
pmb0865	LysoPC 18:3(2n isomer)	6.15E+06	3.02E+06	1.14E+00	-1.02E+00	down
ML10195036	3-Dehydrosphinganine	1.02E+04	4.61E+03	1.12E+00	-1.14E+00	down
Lmhp009129	LysoPC 15:0(2n isomer)	8.67E+03	3.90E+03	1.15E+00	-1.15E+00	down
YC512118	Oleamide (9-Octadecenamide)	2.88E+05	1.29E+05	1.00E+00	-1.16E+00	down
pmb2319	LysoPC 15:0	4.64E+03	2.03E+03	1.06E+00	-1.20E+00	down
pmb2260	LysoPC 15:1	1.31E+04	5.38E+03	1.14E+00	-1.28E+00	down
Lmhp009464	LysoPE 17:1(2n isomer)	1.05E+05	3.44E+04	1.15E+00	-1.62E+00	down

Lmhp008718	LysoPC 17:2	4.06E+04	1.23E+04	1.12E+00	-1.73E+00	down
pmb0881	LysoPE 18:2	8.50E+05	2.26E+05	1.16E+00	-1.91E+00	down
pmb2221	4-Hydroxysphinganine	3.16E+07	7.96E+06	1.17E+00	-1.99E+00	down
Lmhp009590	LysoPC 17:1	4.84E+05	1.17E+05	1.16E+00	-2.05E+00	down
pmp001251	LysoPC 18:2(2n isomer)	1.42E+07	2.83E+06	1.17E+00	-2.33E+00	down
pmb0874	LysoPE 18:2(2n isomer)	8.52E+06	1.04E+06	1.17E+00	-3.04E+00	down
pmb0302	2-Aminoethylphosphonate	3.80E+04	n.d.	1.17E+00	-1.20E+01	down
mws0120	Choline Alfoscerate	8.13E+04	n.d.	1.17E+00	-1.31E+01	down
**Nucleotides and derivatives**						
pmb2507	2-Deoxyribose-1-phosphate	n.d.	4.70E+05	1.17E+00	1.57E+01	up
pme0256	Xanthine	n.d.	2.23E+05	1.17E+00	1.46E+01	up
pme1266	3-Methylxanthine	n.d.	3.48E+04	1.17E+00	1.19E+01	up
pme3968	7-Methylguanine	n.d.	1.95E+04	1.17E+00	1.11E+01	up
pme0264	Thymidine	n.d.	1.91E+04	1.17E+00	1.11E+01	up
pme1109	Guanine	1.16E+04	4.71E+05	1.16E+00	5.34E+00	up
pme3967	2-(Dimethylamino)guanosine	1.63E+04	3.74E+05	1.16E+00	4.52E+00	up
mws0884	Cyclic 3',5'-Adenylic acid	3.57E+03	5.00E+04	1.16E+00	3.81E+00	up
pmb0530	Nicotinic acid adenine dinucleotide	3.43E+04	4.79E+05	1.17E+00	3.81E+00	up
pmb0981	Adenosine 5'-monophosphate	4.15E+05	5.48E+06	1.17E+00	3.73E+00	up

pme3961	2'-Deoxyadenosine	2.27E+04	2.64E+05	1.16E+00	3.54E+00	up
pme3188	Uridine 5'-monophosphate	1.02E+04	8.91E+04	1.16E+00	3.13E+00	up
pmb0374	2-Aminopurine	1.02E+04	6.51E+04	1.16E+00	2.67E+00	up
pme0230	Adenosine	7.94E+06	4.87E+07	1.17E+00	2.62E+00	up
pmb0998	Guanosine 5'-monophosphate	2.18E+04	1.15E+05	1.16E+00	2.40E+00	up
pme2117	Adenosine 5'-diphosphate	1.50E+05	7.09E+05	1.15E+00	2.24E+00	up
mws1060	9-(Arabinosyl)hypoxanthine	1.53E+04	7.20E+04	1.17E+00	2.23E+00	up
mws0248	Uridine	1.62E+04	7.44E+04	1.16E+00	2.20E+00	up
pme0040	Adenine	6.25E+05	2.86E+06	1.17E+00	2.19E+00	up
pme3184	2'-Deoxyadenosine-5'-monophosphate	1.85E+04	8.23E+04	1.15E+00	2.16E+00	up
pme2651	NADP (Nicotinamide adenine dinucleotide phosphate)	1.25E+05	4.18E+05	1.14E+00	1.74E+00	up
pme3337	Succinyladenosine	6.51E+05	2.13E+06	1.13E+00	1.71E+00	up
pme2746	Riboflavin 5'-Adenosine Diphosphate	1.54E+05	4.80E+05	1.17E+00	1.64E+00	up
pme3007	Uridine 5'-diphosphate	1.43E+05	3.79E+05	1.14E+00	1.40E+00	up
pme0183	Isoguanine	1.23E+05	3.00E+05	1.10E+00	1.28E+00	up
ML10180524	Cytarabine	4.57E+04	1.06E+05	1.14E+00	1.22E+00	up
pmb2922	Uridine 5'-diphospho-D-glucose	1.59E+06	3.51E+06	1.15E+00	1.14E+00	up
mws0863	2-Deoxyribose-5'-phosphate	1.33E+05	6.02E+04	1.13E+00	-1.14E+00	down
Lmqp000329	5-Aminoimidazole ribonucleotide	1.87E+05	8.31E+04	1.08E+00	-1.17E+00	down

pmc0274	6-Methylmercaptopurine	4.13E+07	8.73E+06	1.16E+00	-2.24E+00	down
pme1474	5'-Deoxy-5'-(methylthio)adenosine	1.93E+07	1.82E+06	1.17E+00	-3.41E+00	down
mws1641	Lumazine	8.99E+03	n.d.	1.17E+00	-9.96E+00	down
**Organic acids**						
pme3186	DL-Glyceraldehyde-3-phosphate	n.d.	5.56E+05	1.17E+00	1.59E+01	up
pme1975	Malonic acid	n.d.	3.67E+05	1.17E+00	1.53E+01	up
mws0972	6-Hydroxyhexanoic acid	n.d.	2.44E+05	1.17E+00	1.47E+01	up
mws2125	Phosphoenolpyruvate	n.d.	2.20E+05	1.17E+00	1.46E+01	up
Lmgn000242	4,5,6-Trihydroxy-2-oxohexanoic acid	n.d.	5.85E+04	1.17E+00	1.27E+01	up
Lmbn000193	Tartronate semialdehyde	n.d.	2.76E+04	1.17E+00	1.16E+01	up
mws0640	2-Hydroxy-2-methylbutyric acid	n.d.	1.01E+04	1.17E+00	1.01E+01	up
mws0341	2-Hydroxyisocaproic acid	8.63E+03	9.81E+05	1.01E+00	6.83E+00	up
mws0576	3-Hydroxybutyric acid	6.82E+04	4.94E+06	1.17E+00	6.18E+00	up
mws0574	2-Hydroxyisobutyric acid	8.17E+03	1.59E+05	1.17E+00	4.28E+00	up
pme1216	2-Picolinic acid	4.66E+05	6.90E+06	1.17E+00	3.89E+00	up
mws1189	D-Galacturonic acid	5.40E+04	7.01E+05	1.16E+00	3.70E+00	up
mws0470	Methylmalonic acid*	8.01E+05	8.52E+06	1.16E+00	3.41E+00	up
pme3096	Aminomalonic acid	5.87E+05	6.00E+06	1.16E+00	3.36E+00	up
mws0192	Succinic acid*	7.94E+05	8.13E+06	1.16E+00	3.36E+00	up

Lmqp000873	Succinic anhydride	1.63E+05	1.28E+06	1.16E+00	2.97E+00	up
Lmmn003323	2-Hydroxyhexadecanoic acid	6.25E+04	3.99E+05	1.16E+00	2.67E+00	up
Zmgn000503	2,3-Dihydroxy-3-Methylbutanoic Acid	2.57E+05	1.49E+06	1.16E+00	2.53E+00	up
mws0275	L-Malic acid*	4.64E+05	2.24E+06	1.17E+00	2.27E+00	up
Lmbn000198	3-Dehydro-L-Threonic Acid*	7.46E+06	3.58E+07	1.17E+00	2.26E+00	up
pme0048	2-Aminoethanesulfinic acid	2.68E+03	1.24E+04	1.13E+00	2.21E+00	up
pme3011	γ-Aminobutyric acid	1.54E+06	6.66E+06	1.17E+00	2.12E+00	up
pme0295	4-Acetamidobutyric acid	2.21E+04	8.73E+04	1.14E+00	1.98E+00	up
Lmbn001754	3-Isopropylmalic Acid*	4.71E+04	1.61E+05	1.16E+00	1.77E+00	up
mws0346	3-(3-Hydroxyphenyl)-propionic acid	2.48E+03	8.48E+03	1.06E+00	1.77E+00	up
pmb3101	2-Isopropylmalic Acid	4.82E+04	1.58E+05	1.16E+00	1.71E+00	up
Zmgn001448	2-Propylmalic Acid*	5.63E+04	1.68E+05	1.16E+00	1.58E+00	up
pme2380	α-Ketoglutaric acid	1.18E+05	3.28E+05	1.16E+00	1.47E+00	up
Lmmn001643	2-Hydroxycinnamic acid	4.72E+05	1.18E+06	1.17E+00	1.33E+00	up
mws0262	L-Tartaric acid	2.12E+04	5.24E+04	1.13E+00	1.31E+00	up
pme0274	6-Aminocaproic acid	3.20E+06	7.55E+06	1.13E+00	1.24E+00	up
mws0376	Fumaric acid	2.27E+05	5.07E+05	1.11E+00	1.16E+00	up
Lmbp000668	Isonicotinic acid	6.70E+06	1.42E+07	1.17E+00	1.08E+00	up
Zmgn000216	2-Hydroxyethylphosphonic acid	5.14E+04	1.07E+05	1.14E+00	1.05E+00	up

pme3034	Ethylmalonic acid	3.58E+04	1.78E+04	1.10E+00	-1.01E+00	down
pmb2826	L-Citramalic acid	2.77E+05	1.29E+05	1.13E+00	-1.10E+00	down
pme0243	Glutaric acid	8.94E+05	4.02E+05	1.16E+00	-1.15E+00	down
mws0344	D-Xylonic acid	7.16E+06	3.22E+06	1.16E+00	-1.15E+00	down
mws0639	2,3-Dihydroxybenzoic Acid	2.78E+05	1.20E+05	1.05E+00	-1.22E+00	down
Lmbn000216	3-Methylmalic acid*	4.83E+05	1.83E+05	1.15E+00	-1.40E+00	down
Zmyn000247	2-Hydroxyglutaric Acid*	6.35E+05	2.25E+05	1.16E+00	-1.50E+00	down
mws0281	Citric Acid	1.06E+07	3.71E+06	1.17E+00	-1.52E+00	down
Zmyn002323	2-Hydroxyphenylacetic acid	3.93E+04	1.26E+04	1.16E+00	-1.64E+00	down
mws0154	Shikimic acid	1.55E+07	4.93E+06	1.16E+00	-1.65E+00	down
Wmzn000227	2,2-Dimethylsuccinic acid	3.56E+04	1.11E+04	1.05E+00	-1.68E+00	down
Zmgn000217	Methylenesuccinic acid	1.28E+05	3.06E+04	1.16E+00	-2.07E+00	down
Zmyn000453	Isocitric Acid	7.15E+06	1.42E+06	1.17E+00	-2.33E+00	down
mws0277	Quinic Acid	6.36E+06	1.22E+06	1.16E+00	-2.38E+00	down
mws0159	Phenylpyruvic acid	4.80E+05	3.26E+04	1.15E+00	-3.88E+00	down
Zmtn001464	4,8-Dihydroxyquinoline-2-carboxylic acid	6.28E+04	n.d.	1.17E+00	-1.28E+01	down
**Phenolic acids**						
Zmln000668	Monogalloyl-diglucose	n.d.	2.53E+06	1.17E+00	1.81E+01	up
Hmtn001288	Methyl 2,4-dihydroxyphenylacetate	n.d.	6.48E+05	1.17E+00	1.61E+01	up

pme0310	Ethyl gallate	n.d.	3.41E+05	1.17E+00	1.52E+01	up
pmn001533	2-O-Di-gallic acyl-Glucoside-Glucoside	n.d.	1.57E+05	1.17E+00	1.41E+01	up
mws1078	Anthranilic Acid	n.d.	1.27E+05	1.17E+00	1.38E+01	up
NK10253223	2-Amino-3-methoxybenzoic acid	n.d.	9.17E+04	1.17E+00	1.33E+01	up
mws0014	Ferulic acid	n.d.	7.80E+04	1.17E+00	1.31E+01	up
pmb3055	p-Coumaroylquinic acid-4'-O-glucuronide	n.d.	7.54E+04	1.16E+00	1.30E+01	up
mws2212	Caffeic acid	n.d.	6.58E+04	1.17E+00	1.28E+01	up
Lmtn003598	3-Prenyl-4-O-glucosyloxy-4-hydroxybenzoic acid	n.d.	1.65E+04	1.17E+00	1.08E+01	up
mws1212	Methyl ferulate	n.d.	1.18E+04	1.17E+00	1.04E+01	up
pmn001548	2-O-Trigalloyl-glucose-glucose	n.d.	1.10E+04	1.17E+00	1.03E+01	up
pmb2620	3,4-Dimethoxycinnamic acid	n.d.	9.20E+03	1.17E+00	1.00E+01	up
Lmrn001951	(S)-2-Hydroxy-3-(4-Hydroxyphenyl) Propanoic Acid	5.33E+03	7.38E+05	1.16E+00	7.11E+00	up
pmb2795	4-Methoxycinnamic acid*	6.66E+02	2.76E+04	1.16E+00	5.37E+00	up
Lmzn001582	5'-Glucosyloxyjasmanic acid	6.75E+03	2.73E+05	1.17E+00	5.34E+00	up
pmb2819	O-Caffeoyl maltotriose	1.25E+04	3.17E+05	1.17E+00	4.67E+00	up
HJN037	4-O-Methylgallic Acid	3.62E+06	5.09E+07	1.16E+00	3.81E+00	up
pme0309	3-O-Methylgallic Acid	3.24E+06	4.13E+07	1.17E+00	3.67E+00	up
pmn001519	Galloyl Methyl gallate	1.41E+06	1.62E+07	1.16E+00	3.52E+00	up
mws1195	p-Coumaric acid methyl ester*	2.66E+03	2.00E+04	1.16E+00	2.91E+00	up

mws0024	Gallic acid	3.02E+06	2.06E+07	1.17E+00	2.77E+00	up
Zmhn000785	Di-O-Glucosylquinic acid	7.18E+05	4.67E+06	1.17E+00	2.70E+00	up
pme3381	3-Hydroxyphenylacetic Acid Methyl Ester	2.90E+03	1.80E+04	1.14E+00	2.64E+00	up
Hmtn001120	5-(2-Hydroxyethyl)-2-O-glucosylphenol	3.04E+05	1.56E+06	1.16E+00	2.36E+00	up
mws0182	p-Hydroxyphenyl acetic acid	1.04E+04	4.94E+04	1.12E+00	2.25E+00	up
pmn001513	Digallic Acid	1.93E+06	8.74E+06	1.10E+00	2.18E+00	up
pme1439	p-Coumaric acid	3.40E+04	1.30E+05	1.17E+00	1.94E+00	up
pmn001382	Isochlorogenic acid A*	7.33E+03	2.80E+04	1.15E+00	1.93E+00	up
pmn001384	Isochlorogenic acid C*	1.00E+04	2.91E+04	1.10E+00	1.54E+00	up
Lmlp002765	Stilbostemin B	1.08E+04	2.91E+04	1.15E+00	1.43E+00	up
pmn001419	1-O-[(E)-p-Cumaroyl]-D-glucose	3.13E+05	8.13E+05	1.11E+00	1.38E+00	up
mws0749	4-Hydroxybenzoic acid	1.97E+05	4.81E+05	1.14E+00	1.29E+00	up
Lmbn002648	α-Hydroxycinnamic Acid	3.60E+05	8.54E+05	1.15E+00	1.25E+00	up
pmb0142	Caffeic aldehyde	1.90E+04	3.85E+04	1.13E+00	1.02E+00	up
pmn001523	Trigallic acid	2.68E+06	1.31E+06	1.02E+00	-1.03E+00	down
Zmhn001883	Vanillic acid-4-O-glucoside	2.38E+05	1.15E+05	1.13E+00	-1.05E+00	down
Hmdp001663	1,2,6-Tri-O-galloyl-glucose	6.89E+04	3.20E+04	1.13E+00	-1.11E+00	down
pmn001529	3,4,5-Tri-O-Galloylshikimic acid	5.06E+04	2.26E+04	1.07E+00	-1.16E+00	down
mws0180	2,5-Dihydroxybenzoic acid; Gentisic Acid*	1.69E+05	7.00E+04	1.09E+00	-1.27E+00	down

mws0183	3,4-Dihydroxybenzoic acid (Protocatechuic acid)*	3.48E+05	1.43E+05	1.07E+00	-1.28E+00	down
Hmbp001276	Gallacetophenone	8.66E+05	3.53E+05	1.14E+00	-1.29E+00	down
pmn001535	1,3,4,6-Tetra-O-Galloyl-D-Glucose	1.30E+05	4.91E+04	1.10E+00	-1.41E+00	down
pme3443	Sinapinaldehyde	4.14E+04	7.91E+03	1.15E+00	-2.39E+00	down
Zmhn002422	1-O-Feruloyl-D-Glucose	5.78E+05	1.04E+05	1.15E+00	-2.48E+00	down
mws0853	Sinapyl alcohol	1.30E+05	6.60E+03	1.16E+00	-4.31E+00	down
Lmgp003989	Dicaffeoylshikimic acid	3.85E+03	n.d.	1.17E+00	-8.74E+00	down
pmb0751	Trans-5-O-(p-Coumaroyl) shikimate	2.18E+04	n.d.	1.17E+00	-1.12E+01	down
NK10264324	1,3,5-Benzenetriol	3.29E+05	n.d.	1.17E+00	-1.52E+01	down
**Tannins**						
Lmyn002788	Methyl gallate	1.57E+06	2.37E+07	1.17E+00	3.92E+00	up
pme0436	Procyanidin B3	2.78E+04	1.20E+05	1.02E+00	2.11E+00	up
mws0836	Procyanidin B1	1.66E+04	6.96E+04	1.01E+00	2.07E+00	up
**Terpenoids**						
pmp000695	Swertiamarin	1.01E+05	1.63E+04	1.13E+00	-2.63E+00	down
**Xanthone**						
pmp001033	Isomangiferin	2.18E+05	4.94E+04	1.04E+00	-2.14E+00	down
pmf0516	Mangiferin	7.20E+05	1.04E+05	1.04E+00	-2.79E+00	down
**Vitamin**						

pme1306	Pyridoxine-5'-phosphate	n.d.	1.29E+04	1.17E+00	1.05E+01	up
pme1383	Pyridoxine	5.23E+05	2.12E+07	1.17E+00	5.34E+00	up
pmb0789	Pyridoxine-5'-O-glucoside	5.13E+04	3.57E+05	1.17E+00	2.80E+00	up
Zmjp000624	Pyridoxal	2.25E+04	7.90E+04	1.15E+00	1.81E+00	up
mws0232	Riboflavin (Vitamin B2)	3.94E+04	1.18E+05	1.17E+00	1.59E+00	up
pmb0801	4-Pyridoxic acid-O-glucoside	4.89E+04	1.17E+05	1.17E+00	1.26E+00	up
pme0490	Nicotinic acid (Vitamin B3)	6.67E+06	1.42E+07	1.17E+00	1.09E+00	up
pma3101	Nicotinate D-ribonucleoside	3.47E+05	1.61E+05	1.13E+00	-1.11E+00	down
mws1337	D-Pantothenic Acid	3.66E+06	1.67E+06	1.15E+00	-1.13E+00	down
MA10039492	Dehydroascorbic acid	1.57E+06	5.30E+05	1.15E+00	-1.57E+00	down
Hmfn000531	L-Ascorbic acid (Vitamin C)	1.54E+07	4.24E+06	1.17E+00	-1.86E+00	down
pme2596	4-Pyridoxic acid	7.82E+04	1.86E+04	1.17E+00	-2.07E+00	down
mws0133	Nicotinamide	3.07E+06	5.06E+05	1.17E+00	-2.60E+00	down
**Saccharides and Alcohols**						
Zmgn000447	3-Phospho-D-glyceric acid	n.d.	7.84E+06	1.17E+00	1.97E+01	up
pme3705	D-Glucoronic acid	n.d.	7.74E+05	1.17E+00	1.64E+01	up
pme3311	D-Fructose-1,6-biphosphate	n.d.	2.53E+05	1.17E+00	1.48E+01	up
Zmgn000173	D-Ribose	2.42E+04	3.02E+05	1.16E+00	3.64E+00	up
mws1155	Mannitol*	1.08E+04	1.06E+05	1.17E+00	3.29E+00	up

pme2237	Dulcitol*	4.04E+04	3.68E+05	1.16E+00	3.19E+00	up
pme2435	L-Fucose	1.27E+04	7.99E+04	1.15E+00	2.65E+00	up
Zmzn000079	D-Erythrose-4-phosphate	3.65E+04	1.76E+05	1.14E+00	2.27E+00	up
Zmpn000095	Sorbitol-6-phosphate	1.33E+04	4.35E+04	1.17E+00	1.71E+00	up
mws2523	Trehalose 6-phosphate	5.32E+04	1.55E+05	1.17E+00	1.54E+00	up
mws1090	Glucose-1-phosphate*	2.06E+06	5.58E+06	1.17E+00	1.44E+00	up
pme3313	D-Fructose 6-phosphate*	1.11E+06	2.92E+06	1.17E+00	1.40E+00	up
mws0866	D-Glucose 6-phosphate*	2.52E+06	6.64E+06	1.17E+00	1.40E+00	up
Zmyn000110	D-Glucosamine 1-phosphate	7.38E+03	1.84E+04	1.11E+00	1.32E+00	up
mws4175	D-Glucurono-6,3-lactone	1.74E+05	6.65E+04	1.14E+00	-1.39E+00	down
pmb0786	D-Glucosamine	4.76E+06	1.62E+06	1.17E+00	-1.56E+00	down
mws1589	D-Panose	3.84E+05	1.13E+05	1.06E+00	-1.76E+00	down
ML10171848	D-Arabinono-1,4-lactone	4.20E+05	8.74E+04	1.17E+00	-2.27E+00	down
Lmmn000214	Solatriose	5.50E+05	9.39E+04	1.16E+00	-2.55E+00	down
mws4163	Nystose	3.16E+04	n.d.	1.17E+00	-1.18E+01	down
**Others**						
Lmmn003088	2,6-Dimethoxyhydroquinone-1-O-glucoside	n.d.	9.44E+05	1.17E+00	1.67E+01	up
pmp001282	Propyl 2-(trimethylammonio)ethyl phosphate	n.d.	4.19E+05	1.17E+00	1.55E+01	up
mws1038	Pantetheine	1.25E+04	1.04E+05	1.15E+00	3.05E+00	up

Lmmp003215	Icariside B2	7.85E+05	2.17E+06	1.16E+00	1.47E+00	up
Lmmp004382	3-Oxo-α-ionol-β-D-glucoside	1.52E+05	3.88E+05	1.16E+00	1.35E+00	up
pmn001423	Roseoside	2.22E+04	5.27E+04	1.12E+00	1.25E+00	up

n.d.: not detectable; *: isomers

Table S5. The classification of volatile compounds detected by the metabolome in TR *vs.* MG

Compounds	TR	MG	VIP	p_value	Log2FC	Type
**Ester**						
2(3H)-Furanone, 5-ethyldihydro-	3.72E+05	n.d.	1.13E+00	1.60E-02	1.53E+01	up
N-(2,5-ditrifluoromethylbenzoyl)-D-Alanine, pentyl ester	7.60E+04	n.d.	1.13E+00	1.22E-02	1.30E+01	up
Octanoic acid, ethyl ester	5.64E+04	n.d.	1.13E+00	7.98E-03	1.26E+01	up
Butanoic acid, butyl ester	2.80E+04	n.d.	1.13E+00	1.43E-03	1.16E+01	up
Butanoic acid, hexyl ester	2.64E+04	n.d.	1.13E+00	2.89E-03	1.15E+01	up
Ethyl tridecanoate	1.58E+04	n.d.	1.13E+00	2.71E-03	1.08E+01	up
Dodecanoic acid, propyl ester	7.71E+03	n.d.	1.13E+00	1.09E-02	9.74E+00	up
Butanoic acid, 2-methylpropyl ester	7.48E+03	n.d.	1.13E+00	2.59E-03	9.70E+00	up
Ethyl 9-tetradecenoate	7.05E+03	n.d.	1.13E+00	1.47E-02	9.61E+00	up
Hexanoic acid, ethyl ester	6.23E+03	n.d.	1.13E+00	1.07E-02	9.44E+00	up
Tetradecanoic acid, propyl ester	6.17E+03	n.d.	1.13E+00	1.76E-02	9.42E+00	up
Propanoic acid, 2-methyl-, octyl ester	5.43E+03	n.d.	1.13E+00	8.38E-04	9.24E+00	up
Fenobucarb	3.56E+03	n.d.	1.13E+00	2.38E-02	8.63E+00	up
Hexadecanoic acid, propyl ester	3.01E+03	n.d.	1.13E+00	1.78E-02	8.39E+00	up
Dodecanoic acid, ethyl ester	1.27E+06	4.59E+03	1.11E+00	1.10E-02	8.12E+00	up

(6,6-dimethyl-2-bicyclo[3.1.1]hept-2-enyl)methyl 2-methylpropanoate	2.47E+03	n.d.	1.13E+00	1.63E-02	8.10E+00	up
n-Butyl laurate	2.06E+03	n.d.	1.13E+00	2.21E-02	7.84E+00	up
Tetradecanoic acid, ethyl ester	1.34E+06	1.02E+04	1.11E+00	1.28E-02	7.04E+00	up
Decanoic acid, ethyl ester	2.51E+05	1.95E+03	1.13E+00	1.24E-02	7.01E+00	up
Hexadecanoic acid, ethyl ester	6.48E+05	1.09E+04	1.09E+00	1.30E-02	5.90E+00	up
Butanoic acid, phenylmethyl ester	5.71E+04	2.00E+03	1.13E+00	2.10E-02	4.83E+00	up
Methyl tetradecanoate	1.81E+04	1.14E+03	1.13E+00	7.91E-03	3.99E+00	up
Pentadecanoic acid, 14-methyl-, methyl ester	1.99E+04	3.46E+03	1.12E+00	7.35E-03	2.52E+00	up
Ethane, isocyanato-	6.49E+03	2.10E+03	1.08E+00	1.06E-03	1.63E+00	up
Propanoic acid, 2-methyl-, 1-(1,1-dimethylethyl)-2-methyl-1,3-propanediyl ester	1.28E+04	5.98E+03	1.09E+00	1.08E-03	1.09E+00	up
Myrtenyl 3-methylvalerate	3.16E+03	1.20E+04	1.12E+00	9.24E-03	-1.93E+00	down
5-Azulenemethanol, 1,2,3,4,5,6,7,8-octahydro-.alpha.,.alpha.,3,8-tetramethyl-, acetate, [3S-(3.alpha.,5.alpha.,8.alpha.)]-	4.48E+03	5.58E+04	1.13E+00	1.70E-02	-3.64E+00	down
β-Phenylethyl butyrate	4.20E+04	2.30E+06	1.13E+00	8.18E-04	-5.78E+00	down
**Heterocyclic compound**						
(5R,8aR)-5-Propyloctahydroindolizine	4.46E+05	2.64E+03	1.11E+00	2.27E-02	7.40E+00	up

2-Propanamine, 2-methyl-N2-[1-tetrahydro-1H-1-pyrrolylmethylidene]	1.73E+04	4.17E+04	1.13E+00	7.95E-04	-1.27E+00	down
Benzofuran, 4,7-dimethyl-	1.10E+04	2.81E+04	1.10E+00	2.78E-02	-1.36E+00	down
2,2',5,5'-tetrahydro-2,2'-Bifuran	1.21E+05	3.15E+05	1.11E+00	4.90E-03	-1.39E+00	down
7,9-Di-tert-butyl-1-oxaspiro(4,5)deca-6,9-diene-2,8-dione	1.31E+03	3.53E+03	1.05E+00	4.18E-02	-1.43E+00	down
1H-Pyrazole-4-carbonitrile	5.78E+03	3.45E+04	1.11E+00	1.92E-02	-2.58E+00	down
diethylboryl-.delta.-Valerolactam	5.54E+03	2.02E+05	1.13E+00	2.99E-03	-5.18E+00	down
trans-Linalool oxide (furanoid)	4.69E+03	2.10E+05	1.13E+00	8.12E-03	-5.49E+00	down
1-Pentanone, 1-(2-furanyl)-	n.d.	3.29E+03	1.13E+00	3.63E-03	-8.51E+00	down
**Ketone**						
2H-Pyran-2-one, tetrahydro-6-propyl-	1.31E+04	n.d.	1.13E+00	2.22E-02	1.05E+01	up
1-Pentanone, 1-(4-methylphenyl)-	3.03E+04	1.81E+05	1.12E+00	2.09E-02	-2.57E+00	down
Acetophenone, 4'-hydroxy-	3.41E+03	1.34E+05	1.13E+00	3.66E-03	-5.29E+00	down
**Hydrocarbons**						
cis-3,4-diethenyl-Cyclohexene	7.41E+03	4.46E+04	1.13E+00	4.80E-03	-2.59E+00	down
1,4,7,-Cycloundecatriene, 1,5,9,9-tetramethyl-, Z,Z,Z-	1.51E+04	1.86E+05	1.13E+00	7.70E-03	-3.62E+00	down
**Terpenoids**						
2,6-Octadienal, 3,7-dimethyl-, (E)-	4.05E+04	9.35E+04	1.09E+00	3.03E-02	-1.21E+00	down
γ-Terpinene	5.63E+04	1.64E+05	1.12E+00	1.78E-02	-1.54E+00	down

Dicyclopentadiene	1.02E+04	3.09E+04	1.08E+00	2.22E-02	-1.61E+00	down
(+)-2-Bornanone	2.84E+03	1.11E+04	1.13E+00	5.86E-03	-1.97E+00	down
p-Mentha-1,5,8-triene	3.30E+04	1.32E+05	1.13E+00	8.36E-03	-2.00E+00	down
Bicyclo[3.1.1]hept-2-ene, 3,6,6-trimethyl-	2.34E+04	1.06E+05	1.13E+00	9.43E-03	-2.18E+00	down
1,5-Cyclooctadiene, 3,4-dimethyl-	8.01E+04	4.28E+05	1.13E+00	1.24E-02	-2.42E+00	down
β-Phellandrene	1.20E+05	6.92E+05	1.12E+00	6.86E-03	-2.52E+00	down
β-Myrcene	3.74E+04	2.98E+05	1.13E+00	1.69E-02	-2.99E+00	down
(+)-4-Carene	1.13E+06	1.06E+07	1.13E+00	5.23E-03	-3.23E+00	down
3-Carene	4.30E+05	5.08E+06	1.13E+00	1.43E-02	-3.56E+00	down
Caryophyllene	6.71E+03	1.12E+05	1.13E+00	1.18E-02	-4.07E+00	down
(2S,4aR,8aR)-4a,8-Dimethyl-2-(prop-1-en-2-yl)-1,2,3,4,4a,5,6,8a-octahydronaphthalene	3.84E+03	8.04E+04	1.13E+00	1.36E-02	-4.39E+00	down
Naphthalene, decahydro-4a-methyl-1-methylene-7-(1-methylethenyl)-, [4aR-(4a.alpha.,7.alpha.,8aβ)]-	3.75E+04	7.91E+05	1.13E+00	1.01E-02	-4.40E+00	down
2-Isopropenyl-4a,8-dimethyl-1,2,3,4,4a,5,6,8a-octahydronaphthalene	5.67E+03	1.33E+05	1.13E+00	1.71E-02	-4.55E+00	down
3-methyl-6-(1-methylethylidene)-Cyclohexene	8.31E+03	2.00E+05	1.13E+00	4.35E-03	-4.59E+00	down
2-Buten-1-one, 1-(2,6,6-trimethyl-1,3-cyclohexadien-1-yl)-, (E)-	1.41E+04	5.61E+05	1.13E+00	2.11E-03	-5.32E+00	down
3,7,11,15-Tetramethyl-2-hexadecen-1-ol	n.d.	2.23E+03	1.13E+00	1.52E-02	-7.96E+00	down

[1S-(1.alpha.,4β,5.alpha.)]-4-methyl-1-(1-methylethyl)-Bicyclo[3.1.0]hexan-3-one	n.d.	6.50E+03	1.13E+00	9.48E-03	-9.50E+00	down
Naphthalene, 1,2,3,5,6,7,8,8a-octahydro-1,8a-dimethyl-7-(1-methylethenyl)-, [1R-(1.alpha.,7β,8a.alpha.)]-	n.d.	2.05E+04	1.13E+00	6.29E-03	-1.12E+01	down
1,3-Dimethyl-5-(propen-1-yl)adamantane	n.d.	2.22E+04	1.13E+00	5.10E-03	-1.13E+01	down
(1S,4aS,7R,8aS)-1,4a-Dimethyl-7-(prop-1-en-2-yl)decahydronaphthalen-1-ol	n.d.	2.67E+04	1.13E+00	9.56E-03	-1.15E+01	down
**Acid**						
Undecylenic Acid	3.22E+03	n.d.	1.13E+00	9.36E-03	8.48E+00	up
Butanoic Acid, 3-methylbutyl ester	6.84E+04	4.63E+03	1.13E+00	2.11E-03	3.88E+00	up
**Aldehyde**						
BenzAldehyde, 3-ethyl-	2.26E+04	4.41E+03	1.12E+00	2.04E-02	2.36E+00	up
Nonanal	4.44E+04	1.91E+04	1.12E+00	1.26E-02	1.22E+00	up
6-Octenal, 7-methyl-3-methylene-	7.34E+03	2.76E+04	1.11E+00	4.71E-03	-1.91E+00	down
4-Isopropylcyclohexa-1,3-dienecarbAldehyde	5.38E+03	1.25E+05	1.13E+00	1.39E-02	-4.53E+00	down
**Halogenated hydrocarbons**						
Difluorophosphoric acid	2.36E+04	6.75E+03	1.12E+00	1.31E-02	1.80E+00	up
**Phenol**						
2-(1,1-Dimethylethyl)-6-(1-methylethyl)phenol	1.03E+06	4.07E+05	1.11E+00	2.21E-02	1.33E+00	up

Phenol, 2,4,6-tris(1-methylethyl)-	7.06E+03	2.24E+04	1.11E+00	1.55E-03	-1.67E+00	down
8,9-Dehydrothymol	n.d.	1.90E+04	1.13E+00	1.06E-02	-1.10E+01	down
**Aromatics**						
Toluene	3.53E+04	1.29E+04	1.08E+00	3.69E-02	1.45E+00	up
1-ethoxy-4-ethyl-Benzene	8.04E+03	2.85E+04	1.12E+00	1.33E-03	-1.83E+00	down
Benzene, 1-methyl-3-(1-methylethyl)-	2.08E+05	9.06E+05	1.13E+00	6.52E-03	-2.12E+00	down
Benzene, 1-ethenyl-3,5-dimethyl-	6.26E+05	3.08E+06	1.13E+00	1.78E-02	-2.30E+00	down
Formamide, N-phenyl-	5.73E+03	3.40E+04	1.11E+00	1.47E-02	-2.57E+00	down
Benzene, 1-methoxy-4-methyl-2-(1-methylethyl)-	n.d.	5.96E+03	1.13E+00	9.17E-03	-9.37E+00	down
**Alcohol**						
(S,Z)-2-Methyl-6-(p-tolyl)hept-2-en-1-ol	6.74E+03	1.77E+03	1.12E+00	1.45E-02	1.93E+00	up
3,4,5-Trifluorobenzyl Alcohol, 2-methylpropyl ether	2.50E+03	1.32E+04	1.11E+00	3.60E-03	-2.40E+00	down

n.d.: not detectable

Table S6. Significantly differentially expressed structural genes of aliphatic acid pathway in TR *vs.* MG

Gene ID	MG-1_fpkm	MG-2_fpkm	MG-3_fpkm	TR-1_fpkm	TR-2_fpkm	TR-3_fpkm	Log2FC	p-value	padj	Regulated
**ALDH**										
Cluster-16416.79337	0	0	0.12	0.53	0.82	0.65	4.541479008	7.57648E-05	0.000217246	up
Cluster-16416.48910	23.41	24.89	21.55	97.9	95.76	94.18	2.485673039	0	0	up
Cluster-16416.32532	2.41	2.71	0.89	10.96	3.64	3.52	2.045097042	0.000262254	0.000701042	up
Cluster-16416.49904	0.76	0.59	0.25	1.34	1.65	0.64	1.626325873	0.004490663	0.009913449	up
Cluster-16416.74945	3.13	5.65	7.5	9.43	9.74	12.41	1.401955583	2.68531E-06	9.22009E-06	up
Cluster-16416.78913	0.35	0.19	0.57	0.44	0.66	0.61	1.059951329	0.020673153	0.039724062	up
Cluster-16416.82921	2.18	3.51	2.73	0.95	0.94	1.08	-1.055120073	0.00040433	0.001054245	down
Cluster-16416.28747	11.48	13.3	11.41	2.88	4.17	4.68	-1.184499911	8.84608E-10	4.37858E-09	down
Cluster-16416.73372	36.9	31.66	34.1	12.88	11.5	8.52	-1.200574394	1.28197E-12	7.96565E-12	down
Cluster-16416.684	4.34	2.67	3.82	0.82	1.72	1	-1.222685172	0.021596939	0.041287932	down
Cluster-16416.30981	6.58	7.34	7.48	1.92	2.18	2.29	-1.307416056	3.44789E-20	3.25353E-19	down
Cluster-16416.37459	46.59	45.06	45.34	11.13	12.95	13.55	-1.423243164	9.63893E-73	3.00866E-71	down
Cluster-16416.87432	0.85	0.6	0.7	0.08	0.28	0.04	-1.981957084	0.006276177	0.013506494	down
Cluster-16416.59494	14.13	12.66	16.89	2.97	2.21	2.43	-2.080448525	1.37872E-30	1.87681E-29	down

Cluster-16416.69559	1.32	0.61	0.95	0.15	0	0.3	-2.224319025	0.019858199	0.03830151	down
Cluster-16416.65504	0.29	0.45	0.42	0.08	0.08	0.02	-2.231893227	0.000199587	0.000541522	down
Cluster-16416.89708	10.05	10.33	11.03	1.89	1.46	1.52	-2.241284869	1.07284E-46	2.16688E-45	down
Cluster-16416.102670	1.86	2.61	1.14	0.17	0.6	0.09	-2.243500185	0.000754002	0.001892101	down
Cluster-16416.51300	4.6	7.78	2.54	0.69	1.31	0.27	-2.266447107	0.000375917	0.000984274	down
Cluster-16416.91517	1.02	0.92	1.54	0.14	0.18	0.19	-2.30146161	4.66875E-06	1.56115E-05	down
Cluster-16416.78738	4.59	2.91	2.69	0.64	0	0.23	-3.061548812	0.000812668	0.002029349	down
Cluster-16416.69053	127.32	122.35	128.04	10.63	9.74	12.29	-3.09021525	0	0	down
Cluster-16416.10341	1.21	0.97	0.84	0.08	0.07	0.08	-3.314340735	2.37907E-08	1.02765E-07	down
Cluster-20561.0	1.23	1.17	0.82	0	0.12	0.06	-3.765432648	0.000445034	0.001152764	down
Cluster-16416.97978	9.71	11.09	10.19	1.25	0.23	0	-3.952421727	8.98151E-06	2.9055E-05	down
Cluster-16416.11951	0.66	1.42	0.81	0	0.13	0	-4.062999173	0.000420557	0.001093404	down
Cluster-16416.12977	2.74	2.43	3.6	0	0.17	0.09	-4.67908433	1.39194E-06	4.94839E-06	down
Cluster-16416.33559	0.67	0.78	0.85	0.07	0	0	-4.726516263	7.34497E-05	0.000210884	down
Cluster-16416.78653	1.07	0.94	1.12	0	0.06	0	-5.257944612	0.000107862	0.000303516	down
Cluster-16416.12974	0.38	0.93	1.34	0	0	0	-6.283999687	5.20449E-05	0.000152439	down
Cluster-13179.0	1.4	1.41	1.29	0	0	0	-7.526049735	1.48819E-09	7.20463E-09	down
Cluster-16416.65505	0.46	0.81	0.27	0	0	0	-7.615500399	2.18351E-08	9.4699E-08	down
Cluster-16416.61750	1.06	1.23	1.28	0	0	0	-7.822875578	2.27658E-10	1.18841E-09	down

Cluster-16416.12976	2.08	1.71	2.45	0	0	0	-7.837717109	3.38732E-10	1.74375E-09	down
Cluster-16416.90015	4.24	3.11	2.9	0	0	0.02	-8.584891608	1.24409E-12	7.73821E-12	down
Cluster-16416.60131	5.26	6.44	7.2	0	0	0.01	-9.474201299	2.11752E-15	1.5856E-14	down
**CYP**										
Cluster-16416.57754	0.06	0.09	0.06	843.2	642.01	664.78	13.72987615	4.6699E-132	2.8265E-130	up
Cluster-16416.58599	112.75	105.98	121.9	1168.59	1082.59	1202.3	3.781158724	0	0	up
Cluster-16416.54854	0	0.17	0.22	1.89	0.48	0.33	3.21760524	0.005133187	0.01122981	up
Cluster-16416.66749	0	0.09	0.16	0.47	0.73	0.45	3.174075483	0.000555357	0.001419295	up
Cluster-16416.58391	5.44	4.26	5.04	25	26.67	25.22	2.827124772	2.961E-168	2.467E-166	up
Cluster-16416.59802	2.49	3.75	2.28	9.45	10.75	9.89	2.260785612	1.11925E-15	8.50681E-15	up
Cluster-16416.7358	0.17	0.44	0.15	0.93	0.71	0.63	2.053186716	0.000741647	0.001862552	up
Cluster-16416.58366	373.9	431.16	381.1	1031.82	942.4	1093.96	1.814677104	4.9385E-119	2.6333E-117	up
Cluster-16416.62728	2.27	1.62	2.8	3.23	3.26	3.97	1.086485753	1.41578E-06	5.02727E-06	up
Cluster-16416.79750	65.8	60.72	77.06	24.4	25.24	28.24	-1.005759236	3.0355E-08	1.29776E-07	down
Cluster-16416.78572	35.93	36.07	29.66	10.65	10.65	15.39	-1.048338555	6.44547E-08	2.65629E-07	down
Cluster-16416.84068	16.91	17.41	15.98	4.71	8.33	4.71	-1.068001852	1.41694E-05	4.46381E-05	down
Cluster-16416.14705	3.17	6.73	5.8	1.16	1.36	2.19	-1.28789192	0.009343606	0.019381184	down
Cluster-16416.20756	62.85	56.82	57.8	4.36	5.58	5.1	-3.132594019	2.9651E-119	1.5857E-117	down
Cluster-16416.29923	36.44	36.42	35.82	2.28	2.48	2.96	-3.383372769	2.91489E-81	1.01023E-79	down

Cluster-16416.94221	0.59	0.92	0.9	0.08	0	0	-4.490514116	0.000342794	0.000902563	down
Cluster-16416.72664	2.07	1.38	0.66	0	0	0.09	-5.140629989	6.89439E-05	0.00019872	down
Cluster-16416.18261	48.02	48.71	51.25	0.88	0.91	0.6	-5.565068839	9.81775E-25	1.11302E-23	down
Cluster-16416.21126	38.62	41.33	38.5	0.24	0.94	0.65	-5.588396761	3.4836E-108	1.6428E-106	down
Cluster-16416.20288	1.03	1.22	0.9	0.05	0	0	-5.665012492	1.68799E-05	5.26523E-05	down
Cluster-16416.21593	24.06	22.77	25.37	0.25	0.37	0.36	-5.761463933	1.38187E-93	5.56157E-92	down
Cluster-16416.80452	0.74	1.55	1.65	0	0	0	-6.055283081	6.81834E-05	0.000196663	down
Cluster-18734.0	0.83	0.18	0.9	0	0	0	-6.170939037	0.000325999	0.000861164	down
Cluster-16416.23114	4.67	5.24	4.57	0.08	0	0	-6.717893579	1.91467E-10	1.00564E-09	down
Cluster-16416.87257	1.77	1.51	1.19	0	0	0	-6.940763275	1.21571E-07	4.86879E-07	down
Cluster-16416.20287	29.4	35.59	34.07	0.13	0.06	0.26	-7.367741464	1.36568E-39	2.34722E-38	down
Cluster-16416.93336	3.05	2.97	4.01	0	0	0.04	-7.4131562	1.39117E-09	6.755E-09	down
Cluster-16416.39931	1.11	2.65	0.95	0	0	0	-7.468903326	7.75595E-08	3.16935E-07	down
Cluster-16416.25037	4.36	4.91	3.73	0	0	0	-7.986966592	9.30817E-11	5.02457E-10	down
Cluster-16416.82295	19.51	16.32	9.71	0	0	0	-8.25439224	1.09086E-10	5.85149E-10	down
Cluster-14998.1	1.96	3.22	2.97	0	0	0	-8.295328419	2.56862E-11	1.44521E-10	down
Cluster-16416.18259	10.11	8.75	9.64	0	0	0	-9.840355823	1.46535E-16	1.16577E-15	down
Cluster-16416.23211	31.07	27.86	32.92	0	0	0	-11.6881087	5.41452E-23	5.72731E-22	down

FAD

Cluster-16416.42734	0.03	0	0	2.64	2.49	2.38	8.237293756	2.24809E-15	1.67969E-14	up
Cluster-16416.91999	0	0	0.04	0.48	0.32	0.44	5.644835137	1.67809E-06	5.91066E-06	up
Cluster-16416.57676	0.76	1.33	0.91	6.18	8.32	7.8	3.349580689	7.91651E-37	1.2737E-35	up
Cluster-16416.81012	1.47	1.09	1.51	9.87	9.02	8.92	3.213675849	1.7694E-101	7.818E-100	up
Cluster-16416.106225	4.8	8.1	6.63	37.99	33.98	37.87	2.932493988	1.3308E-65	3.73767E-64	up
Cluster-16416.58941	121.29	129.98	131.73	714.63	678.45	708.93	2.896755199	0	0	up
Cluster-16416.55440	1.24	1.8	0.68	6.4	8.93	4.74	2.879147099	1.34503E-13	8.96165E-13	up
Cluster-16416.86749	0.84	0.73	0.56	2.97	4.31	3.66	2.791270757	5.53392E-25	6.32357E-24	up
Cluster-16416.55192	84.46	87.91	86.57	452.3	414.64	453.58	2.785598833	0	0	up
Cluster-16416.57677	2.46	1.46	2.73	9.22	13.76	9.49	2.735517886	3.82269E-21	3.75569E-20	up
Cluster-16416.57153	158.69	169.29	157.41	531.85	476.47	521.08	2.096841639	1.7203E-273	2.79E-271	up
Cluster-16416.62217	5.11	6.67	4.62	13.88	15.45	15.47	1.893210344	3.54514E-39	6.03236E-38	up
Cluster-16416.31104	1.18	1.61	0.9	3.42	3.11	3.24	1.834789824	1.60456E-06	5.66471E-06	up
Cluster-16416.48599	3.24	4.31	2.4	7.57	8.73	8.34	1.723415792	5.72279E-09	2.62872E-08	up
Cluster-16416.57152	0.65	1.82	2	5.17	2.42	2.58	1.620939829	0.003178106	0.007205351	up
Cluster-16416.46352	35.54	39.14	36.69	8.98	6.89	5.74	-1.91859232	1.07437E-32	1.54242E-31	down
Cluster-6444.0	4.41	5.08	4.52	0.02	0	0.1	-6.365636723	1.23393E-36	1.97483E-35	down
Cluster-16416.99090	8.08	7.01	6.19	0.12	0	0.04	-6.610937977	2.7857E-18	2.4083E-17	down
Cluster-16416.83617	0.48	0.14	0.98	0	0	0	-6.761659594	3.15764E-05	9.51644E-05	down

Cluster-745.0	13.25	15.9	10.87	0	0	0.16	-7.432381344	1.34347E-09	6.53327E-09	down
Cluster-12825.0	2.93	3.14	1.38	0	0	0	-7.517581471	1.61743E-08	7.11587E-08	down
Cluster-16416.74729	4.11	4.49	4.65	0.04	0	0	-7.720998876	1.44636E-10	7.67781E-10	down
Cluster-16416.4538	4.86	6.24	6.29	0	0	0	-8.900068472	1.78018E-13	1.17747E-12	down
**PDC**										
Cluster-16416.69106+A2095:G7084	12.11	12.78	11.57	73.62	67.87	57.69	2.876755573	6.7867E-90	2.603E-88	up
Cluster-16416.59029	24.6	23.99	23.47	106.71	96.88	96.1	2.496562493	1.0557E-249	1.5065E-247	up
Cluster-16416.53564	15.04	21.82	16	74.41	68.01	62.85	2.400500664	1.84051E-59	4.72933E-58	up
Cluster-16416.76613	0.95	0.39	1.06	3.01	2.51	3.83	2.384818482	1.1282E-09	5.52278E-09	up
Cluster-16416.78703	44.89	40.21	52.85	201.14	163.78	172.77	2.310716858	4.14399E-50	8.95217E-49	up
Cluster-16416.83286	1.5	1.82	1.76	6.7	5.83	5.91	2.289185205	1.5906E-17	1.32785E-16	up
Cluster-16416.57988	107.51	111.02	102.73	393.65	363.06	381.84	2.269330344	0	0	up
Cluster-16416.53427	25.57	20.48	24.06	82.07	73.43	80.98	2.192271308	9.76164E-97	4.07269E-95	up
Cluster-16416.55675	67.13	69.86	64.68	181.92	160.02	176.67	1.808258629	8.9024E-166	7.2513E-164	up
Cluster-16416.49821	78.24	96.45	84.53	217.82	202.61	201.49	1.688730487	2.6148E-103	1.1801E-101	up
Cluster-16416.55676	48.27	49.97	46.61	115.65	112.23	115.14	1.688549196	0	0	up
Cluster-16416.55672	76.25	79.19	76.77	187.82	174.64	183.29	1.676166651	2.9325E-291	5.1869E-289	up
Cluster-16416.73893	0.55	0.69	1.14	2.04	1.8	1.44	1.583091001	1.02179E-05	3.27964E-05	up

Cluster-16416.54396	109.15	123.76	112.59	253.06	239.72	266.49	1.58009268	5.9028E-142	3.92E-140	up
Cluster-16416.45239	0.43	0.61	0.43	1.46	1.2	0.37	1.487496692	0.017414828	0.034054518	up
Cluster-16416.55677	14.25	18.67	14.37	35.35	28.66	32.71	1.480105141	7.72383E-29	1.00296E-27	up
Cluster-16416.54492	117.22	118.48	118.13	188.57	184.64	189.83	1.114178673	0	0	up
Cluster-16416.59722	8.56	6.97	6.54	11.83	10.07	12.08	1.02879175	3.90877E-05	0.000116415	up
**HPL**										
Cluster-16416.40253	1.92	2.76	2.68	0	0	0	-8.728740211	8.5162E-13	5.36175E-12	down
Cluster-16416.75085	169.57	158.69	174.37	0.16	0.28	0.1	-9.400487317	8.6056E-145	5.8792E-143	down
**ADH**										
Cluster-16416.57211	34.02	34.75	31.14	1913.84	1605.26	1472.94	6.076539948	0	0	up
Cluster-16416.45956	0	1.11	0.57	30.9	21.64	13.63	5.756162559	6.37832E-09	2.91429E-08	up
Cluster-16416.24873	0.27	0.39	0.1	3.75	3.98	3.4	4.298827746	1.82605E-11	1.03885E-10	up
**LOX**										
Cluster-16416.58012	165.05	219.48	169.87	6028.25	6152.42	6648.01	5.517285069	0	0	up
Cluster-16416.27899	1.71	2.24	1.96	0	0.06	0	-5.995132903	3.12071E-20	2.95142E-19	down
Cluster-16416.17114	5.53	4.52	4.9	0	0.02	0.03	-7.572407811	3.17311E-30	4.28425E-29	down
Cluster-16416.39017	113.27	113.2	113.92	0.32	0.26	0.37	-8.047632669	2.35E-226	2.8993E-224	down
Cluster-16416.14508	2.44	2.16	2.23	0	0	0	-8.163694943	2.20064E-11	1.24304E-10	down
Cluster-11640.0	1.46	1.84	1.46	0	0	0	-8.355395545	7.31803E-12	4.29471E-11	down

**AAT**										
Cluster-16416.64126	5.3	7.44	7.09	26.07	23.31	30.33	2.454250318	7.38489E-58	1.83966E-56	up
Cluster-16416.49230	27.69	31.62	26.29	65.6	70.26	65.65	1.681699795	3.1283E-112	1.5389E-110	up
Cluster-16416.48227	7.35	7.08	8.4	3.05	1.43	1.81	-1.409574704	3.33014E-07	1.27359E-06	down
Cluster-16416.55974	0.47	0.46	0.48	0.14	0.08	0	-2.261208126	0.011412382	0.023224624	down

Table S7. Expression profiles of same and differentially expressed genes (DEGs) encoding transcription factors (TFs) in TR *vs.* MG

TFs name	No. of TFs*	Upregulate	Downregulate	Biological functions
NAC	116	73	43	Fruit development, plant stress response
WRKY	100	91	9	Defense responses
bHLH	96	89	7	Plant development, substance metabolism
MYB	76	56	20	Cell development, anthocyanin pathway
AP2/ERF	73	64	9	Plant development, stress response
bZIP	31	24	7	Photomorphogenic, fruit ripening
HSF	19	15	4	Plant development, stress response
COP1	18	17	1	Photomorphogenesis and flowering of plant
MADS-box	10	6	4	Fruit development
HY5	1	1	0	
Others	24	4	20	
Total	564	440	124	

* P-adjust value < 0.01 and $|Log_2FC| \geq 2$ as the threshold.

Supplementary Figure legends

Figure S1. Nine quadrant diagram of correlation analysis. From left to right, top to bottom, divided into quadrants 1-9 in order.

Figure S2. Transcriptome and metabolome loading plot analysis.

Figure S3. RT-qPCR validation. Twenty unigenes were selected to validate the RNA-Seq results. Relative expression of the genes in MG and TR samples is shown. Three biological replicates were used.

Figure S1

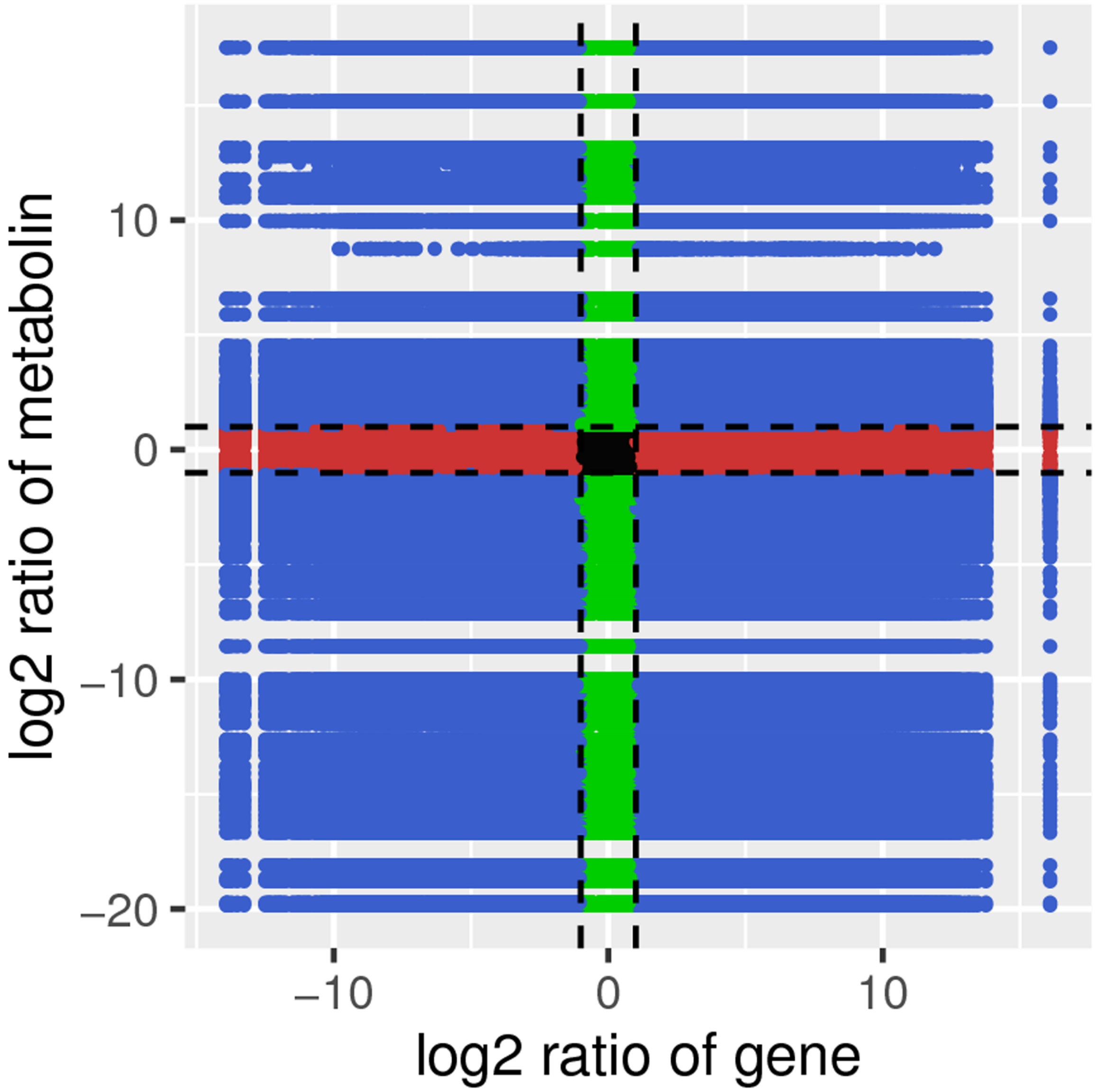

Figure S2

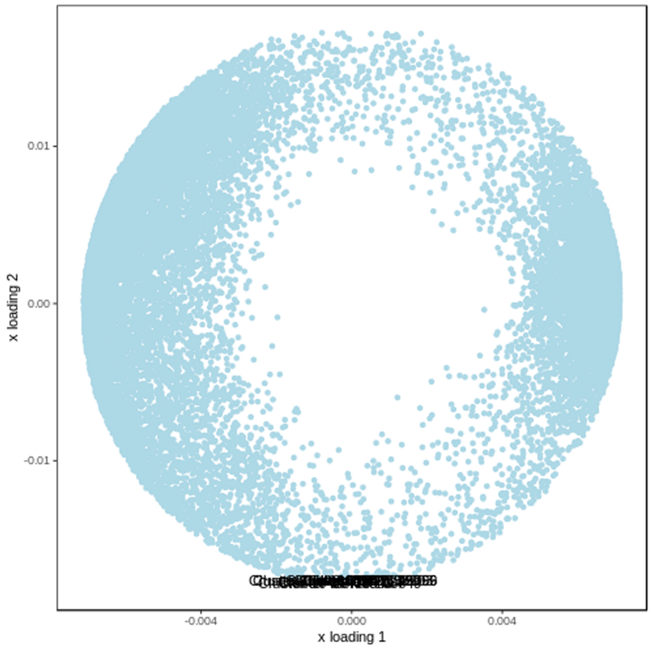

Transcriptome loadings

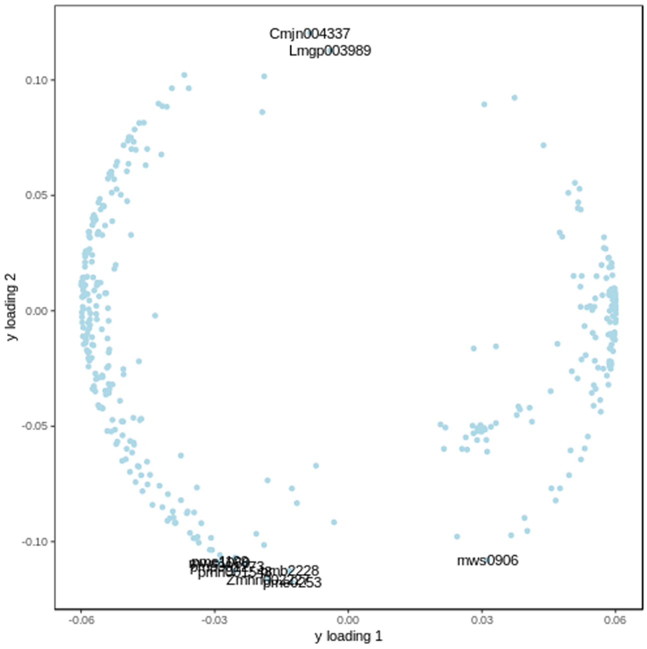

Metabolome loadings

Figure S3

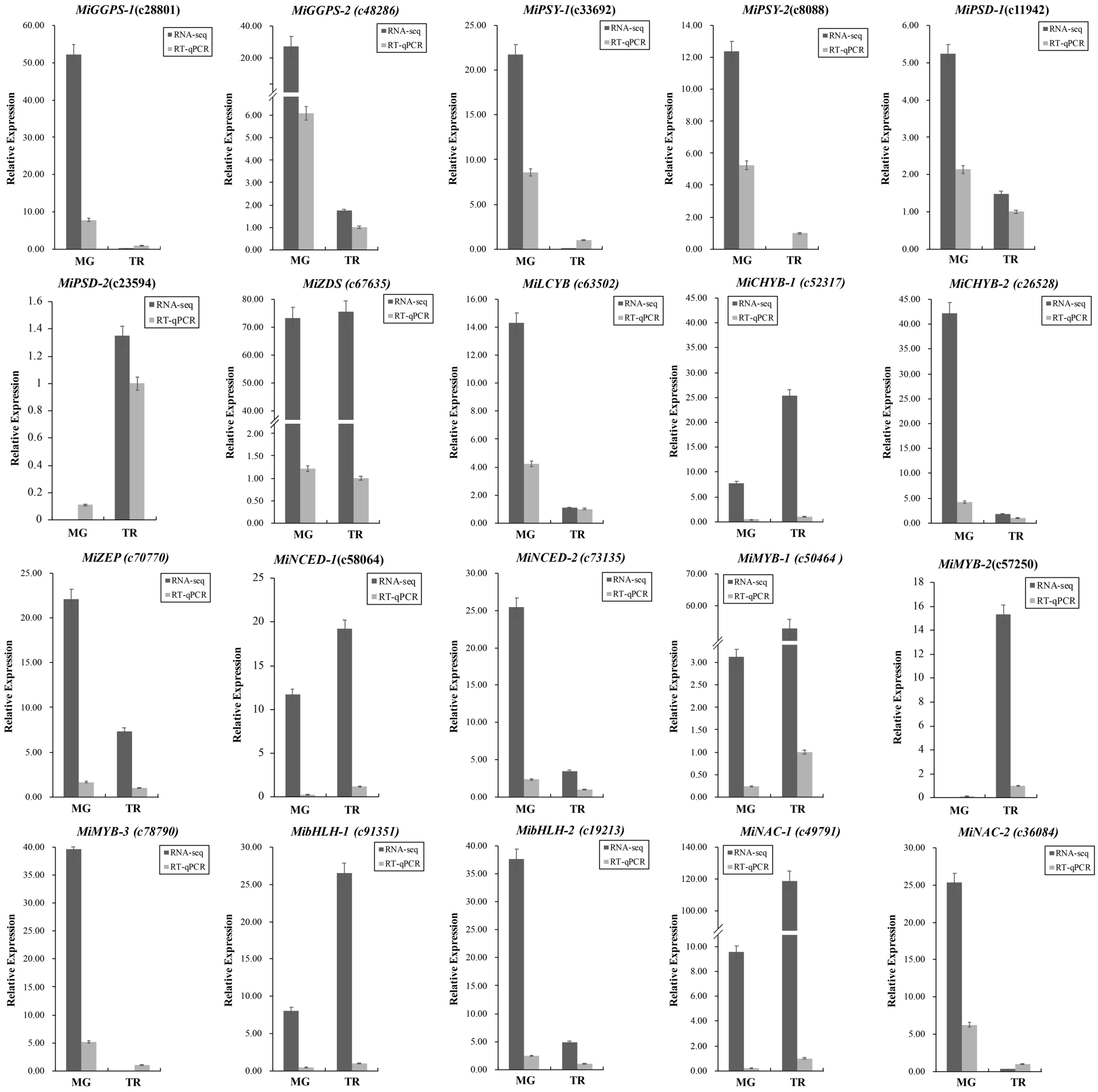
MiGGPS-1(c28801)
MiGGPS-2 (c48286)
MiPSY-1(c33692)
MiPSY-2(c8088)
MiPSD-1(c11942)
MiPSD-2(c23594)
MiZDS (c67635)
MiLCYB (c63502)
MiCHYB-1 (c52317)
MiCHYB-2 (c26528)
MiZEP (c70770)
MiNCED-1(c58064)
MiNCED-2 (c73135)
MiMYB-1 (c50464)
MiMYB-2(c57250)
MiMYB-3 (c78790)
MibHLH-1 (c91351)
MibHLH-2 (c19213)
MiNAC-1 (c49791)
MiNAC-2 (c36084)
RNA-seq
RT-qPCR
Relative Expression
MG
TR
